# Human 14-3-3 Paralogs Differences Uncovered by Cross-Talk of Phosphorylation and Lysine Acetylation

**DOI:** 10.1371/journal.pone.0055703

**Published:** 2013-02-13

**Authors:** Marina Uhart, Diego M. Bustos

**Affiliations:** Laboratorio de Biología Estructural y Celular de Modificaciones post-traduccionales, Instituto de Investigaciones Biotecnológicas-Instituto Tecnológico de Chascomus (IIB-INTECH), Universidad Nacional de San Martín (UNSAM), Consejo Nacional de Investigaciones Científicas y Técnicas (CONICET), Int. Marino Km 8.2, Chascomus, Argentina; Semmelweis University, Hungary

## Abstract

The 14-3-3 protein family interacts with more than 700 different proteins in mammals, in part as a result of its specific phospho-serine/phospho-threonine binding activity. Upon binding to 14-3-3, the stability, subcellular localization and/or catalytic activity of the ligands are modified. Seven paralogs are strictly conserved in mammalian species. Although initially thought as redundant, the number of studies showing specialization is growing. We created a protein-protein interaction network for 14-3-3, kinases and their substrates signaling in human cells. We included information of phosphorylation, acetylation and other PTM sites, obtaining a complete representation of the 14-3-3 binding partners and their modifications. Using a computational system approach we found that networks of each 14-3-3 isoform are statistically different. It was remarkable to find that Tyr was the most phosphorylatable amino acid in domains of 14-3-3 epsilon partners. This, together with the over-representation of SH3 and Tyr_Kinase domains, suggest that epsilon could be involved in growth factors receptors signaling pathways particularly. We also found that within zeta’s network, the number of acetylated partners (and the number of modify lysines) is significantly higher compared with each of the other isoforms. Our results imply previously unreported hidden differences of the 14-3-3 isoforms interaction networks. The phosphoproteome and lysine acetylome within each network revealed post-transcriptional regulation intertwining phosphorylation and lysine acetylation. A global understanding of these networks will contribute to predict what could occur when regulatory circuits become dysfunctional or are modified in response to external stimuli.

## Introduction

Signaling networks regulate all processes within cells using a “language” based on posttranslational modifications (PTMs) of proteins. PTMs represent important regulatory states that, when combined; act as molecular codes to generate a functional diversity beyond genome and transcriptome. Phosphorylation was the first PTM described [1], affecting approximately one third of all proteins in a cell [2]; thus playing a crucial regulatory role in intracellular signal transduction. The first protein kinase activity was observed in 1954 [1]. Twelve years later, Moore and Perez named “14-3-3” an abundant family of proteins, due to its particular elution pattern on two-dimensional DEAE-cellulose chromatography and starch gel electrophoresis [3]. It was not until 30 years later (1996) that it was discovered that interactions of this family with their partners were mediated by phosphoserine or phosphothreonine interaction motifs [4]. Together with kinases and phosphatases, the ubiquitous regulatory 14-3-3 proteins, are essential components of phosphorylation-mediated signaling. It is important to note that the 14-3-3 protein family is functionally different from phospho-binding domains such as WD40, PDZ or WW. Two highly conserved 14-3-3 paralogs are present yeast, seven in mammals, and up to 15 isoforms in plants [5]. These paralogs self-assemble into homo- or hetero-dimers regulating a diverse array of cellular proteins. Several hundreds of 14-3-3 ligands have been reported in low- and high-throughput studies [6]. The 741 14-3-3’s clients studied by MacKintosh [7] largely exceeded the previous estimation made by Jin, J. *et. al* in 2004, that 14-3-3 proteins could theoretical bind the 0.6% of the human proteome [8]. These 14-3-3 partners are involved in diverse processes like regulation of the cytoskeleton, GTPase function, membrane signaling, cell fate determination, response to insulin and TNF-alpha, cell cycle progression and apoptosis [9]. The ability to interact with many different proteins is in part due to their specific phospho-serine/phospho-threonine binding activity. Three high-affinity 14-3-3 binding motifs have been described in 14-3-3 target proteins: RSXpS/TXP (mode 1), RXXXpS/TXP (mode 2) and pS/T-X(1–2)-COOH (mode 3), where pS/T represents phospho-serine/threonine and X is any amino acid [5]. Each 14-3-3 monomer contains an independent ligand-binding channel, thus a dimer can interact with two motifs simultaneously, found either on a single target or on separate binding partners [10]. The latter makes of 14-3-3 dimer a scaffold protein that coordinates the physical assembly of components of a signaling pathway or network [11]. Besides the scaffold role, 14-3-3 dimers are highly rigid structures and binding can induce conformational changes in their protein ligands [12], [13]. This might alter the stability and/or catalytic activity of the ligands [14]. In addition, 14-3-3 binding can hide intrinsic localization motifs, prevent molecular interactions and/or modulate the accessibility of a target protein to modifying enzymes such as kinases, phosphatases or proteases [5], [14]_ENREF_15. Although originally the different isoforms were thought to be redundant, the fact that seven functional paralogs are strictly conserved within all mammals raises questions about their roles and specificity; whether these isoforms have specific or overlapping functions has been argued [15]–[17]. Because of their high conservation, it seems reasonable to suspect that each 14-3-3 isoform has (at least one) distinct functions. Although yeast and human isoforms have failed to reveal any isotype-specific phosphopeptide binding in a full *in vitro* assay [15], there are many reports containing examples of *in vivo* isoform specificity (see Table 1 in [18]). Additionally, some studies have shown tissue and/or cell cycle phase specific expression of 14-3-3 isoforms [17], and specific kinase regulation has been demonstrated [19]. Structural data show little divergence in the phosphopeptide-binding pockets of different 14-3-3s [20], and because most 14-3-3 binding motifs conform to a few consensus sequences, it seems that isoform specificity does not reside in the binding site sequence of the binding partner [21]. Indeed, it most likely depends on additional contacts with the partner protein probably involving residues, such as anchors [22], outside the 14-3-3 binding motifs [21]. Specific 14-3-3 isoforms could be targeted in biomedical treatments for many of the 14-3-3 related diseases that impact humanity world-wide, as many cancers and neurodevelopmental disorders [23].

The extraordinarily high sequence conservation between the seven 14-3-3 mammalian isoforms possesses an important technological challenge to researchers working on these proteins. It was postulated that a systems-level approach is necessary to study protein phosphorylation [2], and we think that, as part of the phosphorylation machinery, to map 14-3-3 network’s components is necessary to understand their functions. It is also important to consider that current literature thoroughly discusses the existence and functional relevance of a phospho-acetylation link [24]–[27]. This makes interesting to integrate the phosphorylation-dependent signaling networks with information about the reversible acetylation of lysine, among others. Lysine acetylation preferentially targets large macromolecular complexes involved in diverse cellular processes, such as chromatin remodeling, regulation of gene expression, cell cycle, splicing, nuclear transport, and actin nucleation. Lysine acetylation is a reversible modification, in contraposition to acetylation of the amino terminus, which appears to be irreversible. The latter occurs in more than 80% of the human proteins and is catalyzed by N-terminal acetyltransferases predominantly during protein synthesis [28]. Lysine acetylation neutralizes its positive charge, and is linked to phosphorylation in various ways. Perhaps the most relevant to mention here is that acetylation impairs phosphorylation-dependent interactions of 14-3-3, either by acetylation of the essential Lys49 (and/or other Lys) on the 14-3-3 binding pocket, or by acetylation of a 14-3-3 partner. Choudhary et al. (2009) showed that analogous sites are acetylated in multiple 14-3-3 isoforms. In their study, acetylation mimetic mutants of 14-3-3 showed impaired binding to synthetic peptides as well as to full-length proteins from whole-cell lysates [29]. These studies uncovered a mechanism that modulates phosphorylation-dependent interactions besides the phosphopeptide-binding domains, and suggest a crosstalk between phosphorylation and lysine acetylation. Both PTMs are very common, they often co-occur within the same protein and are frequently observed at interaction interfaces and in multifunctional proteins [27], [29].

Here, we used the protein interaction network analysis (PINA) platform [30] to construct phosphorylation-14-3-3 protein-protein interaction (PPI) networks corresponding to each 14-3-3 paralog. These networks were compared to search for 14-3-3 isoforms functional and/or spatial differences. Our approach was focused on the analysis of 14-3-3 partner’s domain composition, gene ontology (GO) enrichment and PTMs (phosphorylation, acetylation and others) in general, not specifically orientated to the 14-3-3 binding sites, as in studies by the MacKintosh group [7], [9], [11]. Each polarized isoform network was characterized with respect to those parameters of their nodes, their inherent network features (motifs) and compared. The crosstalk between phosphorylation and lysine acetylation within 14-3-3 isoforms networks was analyzed.

## Methods

### Computer Programming and Statistics

The scripts for the data analysis were programmed with Perl and are freely accessible under request by e-mail to the corresponding author. All statistical analyses to evaluate significance (Wilcoxon rank-sum, Kruskal-Wallis and Fisher’s exact test) were carried out using the R and Rward statistical analysis package. For the analysis of distributions we used the Wilcoxon rank-sum and Kruskal-Wallis, and for those data without distributions we used the Fisher’s exact test. The similarity between paralog networks was assessed by the Jaccard similarity coefficient (Jaccard index).

### 14-3-3-binding Proteins, Kinases and Kinase Substrates

The list of human proteins interacting with each paralog of 14-3-3, kinases and kinase substrates were recovered from the Protein Interaction Network Analysis (PINA) platform [30], which integrates PPI data from six databases. This platform is regularly updated and contains non-redundant integration of data from the following databases: IntAct, MINT, BioGRID, DIP, HPRD and MIPSMPact. The list was also manually revised and curated (using the information of non-interacting proteins from HPRD) and integrated with the PTMs information from HPRD v.10 [31]. This database currently contains information of 16,972 PTMs belonging to various categories such as acetylation, phosphorylation (discriminated by amino acid), dephosphorylation, glycosylation and others, for the majority of the annotated proteins.

Our 14-3-3 full network contain 741 clients (for details see [6]), in agreement with Mackintosh’s work [7] that after applying an ‘inclusion list’ and ‘exclusion list of common contaminants’ identified 750 proteins as 14-3-3 clients. To these 741 proteins network we added the kinases and kinases substrates (and their corresponding edges) resulting in a 2230 nodes and 4870 edges network.

### Disorder Predictions

All disorder predictions were made by using the Cspritz [32] web page (http://protein.bio.unipd.it/cspritz/). Cspritz is an algorithm to detect disordered regions from primary sequence, based on 3 prediction systems to find regions of protein disorder. http://distill.ucd.ie/punch/Punch, which is a Support Vector Machine (SVM) based predictor that utilizes sequence and structural information from homologues. The Spritz, again a SVM based predictor based solely on sequence information, and the ESpritz, based on a machine learning method which does not require sliding windows or any complex source of information.

### Predicting Causal Interactions with a Naïve Bayesian Classifier

To predict edge directions between interacting proteins in our 14-3-3 paralog networks, we used the naïve Bayesian classifier available at Weka v3.4.11 as previously done in [33]. We also acknowledge the training set, instance file and their final directed network to them.

### Network Motif Analysis

To find the network motifs, we used the Fanmod program [34]. This software uses a directed network as input and detects network motifs that occur more often in the real network than in random networks with the same size and connectivity properties. We searched for significantly enriched network motifs with default cutoffs in each 14-3-3 network compared to 1000 random networks. For the triad significance analysis, we used the *p*<0.05 probability value obtained from the Fanmod program.

### Domains and Domain Clubs Analysis

The information of domains contained on each protein used in this analysis was obtained from the HPRD v10 database [31]. All the domains present on each protein of the full network were used for the analysis; however, for clarity, the small and super-numerary coil coil (CC) and transmembrane (TM) domains were omitted in the analysis. These domains were present in all the paralogs with almost no differences between them. The hierarchical domain-based clustering of the proteins encoded by seven Ensembl genomes and the number, composition and architectures of the domain clubs were obtained with kind permission from [35].

### Analysis of Gene Ontology Enrichment within 14-3-3 Isoforms Networks

The Biological Networks Gene Ontology (BiNGO) plugin [36] from Cytoscape [37] was used to determine which Gene Ontology (GO) categories from the files “Biological Process” and “Cellular Component” are statistically over-represented within each 14-3-3 isoform network. BiNGO produces an output file listing the *p*-values of all categories with significant enrichment, maps the predominant functional themes of each 14-3-3 isoform partners on the GO hierarchy, and outputs this mapping as a Cytoscape graph (Fig. S4, S5 & S6). For each isoform, the maximum *p* value was settled in a first analysis to obtain a Cytoscape graph containing between 35 to 50 GOs. On these graphs, each node represents a GO term; the yellow and orange nodes represent terms with significant enrichment, with darker orange representing a higher significance. White nodes are terms with no significant enrichment, but are included because they have a significant child term. Branches of GO with no significant terms are not shown. The size of each node in the BiNGO graph is proportional to the number of nodes in the query set with that term. Because 14-3-3 interaction networks are obviously enriched in phosphorylation, and kinases networks were intentionally added, we highlighted with small stars on the Cytoscape graphs those branch-terminal significantly enriched GOs that are not directly related to kinases or phosphorylation. We repeated the BiNGO analysis but this time *p*<0.05 was settled for all isoforms. Those GO categories marked with a star in any of the Cytoscape graphs from the first analysis were searched on each list (from the second analysis) of all nodes with significant enrichment, and the –log (*p* value) was represented as a bar graph. We repeated the same procedure for the sub-networks of acetylated partners of each isoform. For simplicity, only GOs that showed differences on enrichment between isoforms networks were included in the graphs.

## Results

### The 14-3-3 Isoform’s Networks are Different

**Figure 1:**
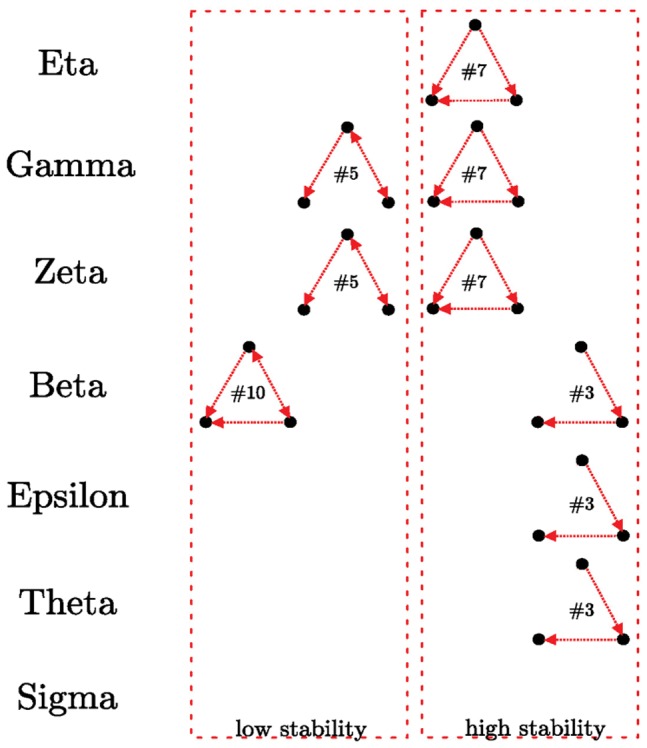
3-nodes statistical significant motifs (*p*<0.005) that built each isoform specific interaction network. The numbers correspond to the classification described in the motif dictionary by Uri Alon (http://www.weizmann.ac.il/mcb/UriAlon/).

The strikingly high conservation of seven 14-3-3 paralogs among all mammals questioned the initial general idea that the different mammalian 14-3-3 isoforms were functionally redundant. Today, a growing number of individual examples evidence some specific roles for them [16]. Beyond this tendency, a comparison of the different isoforms PPI networks was lacking. We used PINA (The Protein Interaction Network Analysis) platform, which includes a database of unified protein–protein interaction data integrated from six manually curated public databases, to create PPI sub-networks for each human 14-3-3 paralog, and a full 14-3-3 signaling network by addition of the seven isoform specific sub-networks. The diverse annotation systems were transcribed so that each human protein was assigned a UniProt [38] identifier code. We also collected all the kinases and experimentally-determined substrates of kinases published in the Human Protein Resource Database (HPRD), and added the phosphorylation sites and other posttranslational modifications (such as lysine acetylation sites) from the same database. This gave us a complete representation at high resolution of the 14-3-3 binding partners and their posttranslational modifications (Table S1). To know the overlapping degree of the 14-3-3 isoform specific networks we compared it by using the Jaccard index (Table S2). This analysis show a low overlapping between the isoform specific networks, the most similar ones being those corresponding to theta and beta isoforms (Jaccard index = 0.273). To study the network properties and 3-nodes motif composition, we transformed the full network into a directed one by using a naïve Bayesian predictor implemented in Weka [39]. The analysis of the directed sub-networks corresponding to each 14-3-3 paralog showed statistical differences in the 3-nodes composition (*p*<0.005, Fig. 1). The motifs in figure 1 are over represented in 14-3-3 sub-networks compared with 1,000 random networks of the same size and degree of distribution. As a reference, in signal transduction networks of *E. coli* and *S. cereviceae* the motifs number 7 and 10 are enriched, and motif number 5 is under represented, among others [40]. The motif number 7 is a feed-forward loop stable motif [41] (*S*tructural *S*tability *S*core, *SSS* = 1) present in eta, gamma and zeta networks; it was initially described as two transcription factors, one of which regulates the other, both jointly regulating a target gene [40], [41]. At the PPI level, this motif could represent the scaffold function, where a protein (in this case 14-3-3) facilitates the interaction between two other proteins (one of them regulates the other one). Gamma and zeta networks are additionally enriched in the unstable motif number 5 (*SSS*∼0.4), which is negatively correlated with signal transduction networks [40]. The unstable motif 10 (*SSS*∼0.4) is exclusively over represented in the beta isoform network (Fig. 1). This motif 10 was described initially as “Interacting transcription factors that co-regulate a third gene” motif [41]. In most pairs of interacting transcription factors that co-regulate genes, the two pair mates are known to have the same function, either co-activating or co-repressing genes [41]. 14-3-3 beta network is also enriched in motif number 3, together with theta and epsilon. This is a stable motif (*SSS* = 1), which is not over-represented in signal transduction networks. Sigma network is the only one with no over representation of any particular motif.

**Figure 2:**
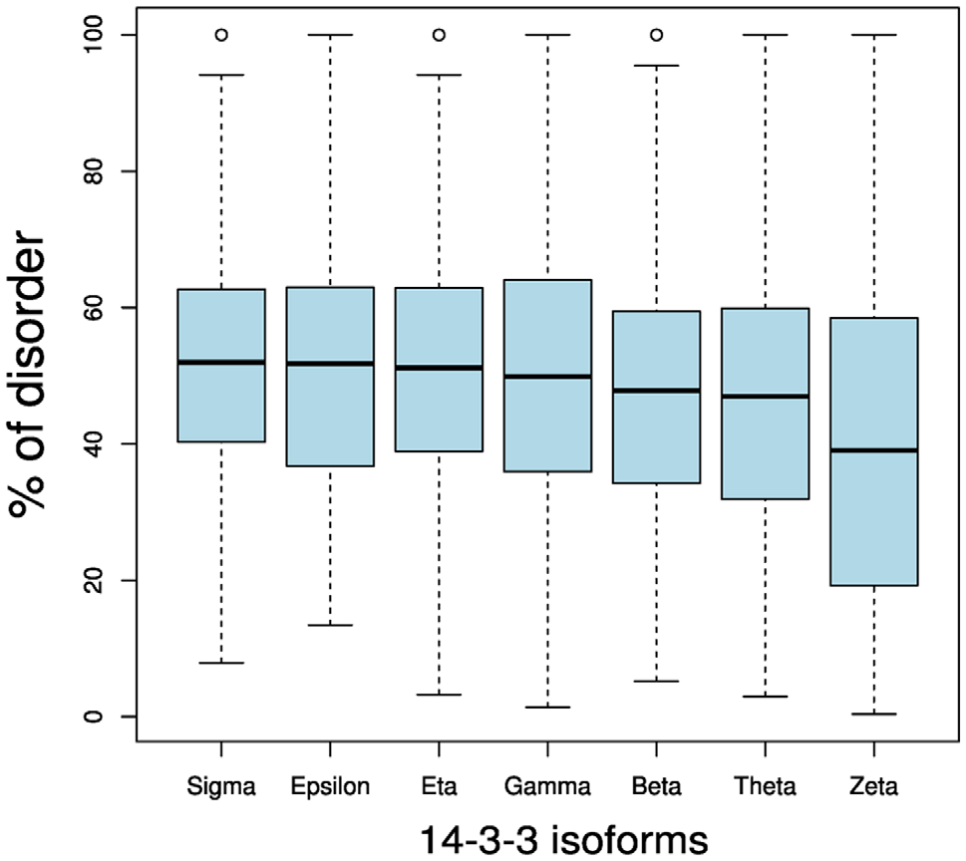
Box plot representation of the disorder content in partners of each 14-3-3 family member. Fisher exact test comparisons between isoforms are summarized in the Table S3.

**Figure 3:**
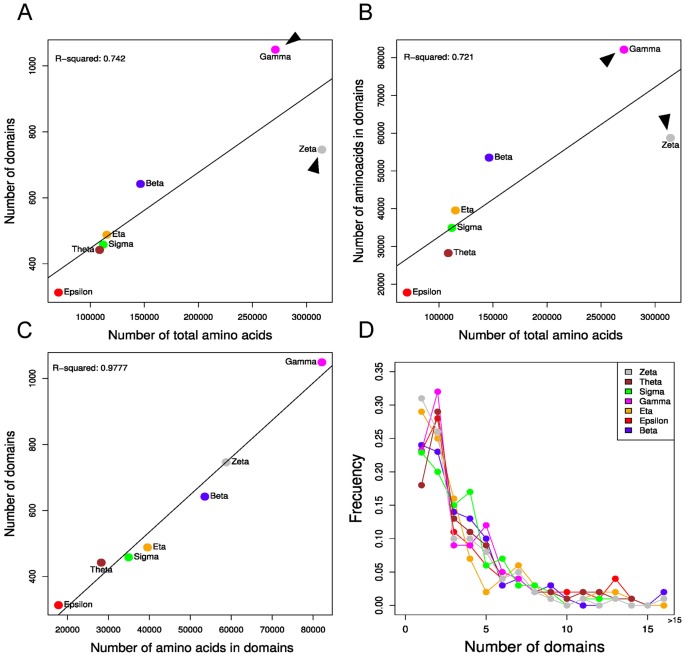
Domain abundance of 14-3-3 isoforms’ partners. A) Number of domains and B) number of amino acids in domains *vs* number of total amino acids. C) Number of domains vs number of amino acids in domains. D) Frequency of partners with x numbers of domains within each 14-3-3 isoform network. The colors for each 14-3-3 isoform are maintained throughout the different figures.

Another feature that shows differences between each isoform specific network is the intrinsic disorder content [42]. A large proportion of the 14-3-3’s interactome is intrinsically disordered [6] which has strong repercussions in its biochemistry [43]. Our previous analyses have revealed that 14-3-3 binding sites are contained in disordered regions, and that 14-3-3 partners are highly disordered, promoting a densely interconnected network [12]. Figure 2 shows a boxplot representation of the disorder content in partners of each 14-3-3 family member. Kruskal-Wallis analysis showed a statistically significant difference in the structural disorder content of specific paralogs partners (*p* = 2.044 e^−09^). In particular, partners of zeta isoform are less disordered than partners from all the other isoforms (5.86 e^−08^≤*p*≤2.53 e^−03^, Fisher exact test). All the results of the Wilcoxon rank sum test from the comparisons between isoforms are summarized in Table S3. These differences in percentage of intrinsical disorder could reflect differences in the domain number and composition of 14-3-3 isoforms’ partners. Using the last version of HPRD database we annotated the domains of each partner of 14-3-3 and analyze the size, number and co-appearance. As shown in figure 3, the number of domains (Fig. 3A) or number of amino acids in domains (Fig. 3B) of sigma, epsilon, eta, theta and beta networks are directly proportional to the total number of amino acids. This means that more partners, or bigger partners, have a larger number of domains. However, the isoforms gamma and zeta have more and less domains (or amino acids in domains) respectively per total number of amino acids (Fig. 3A & B). This could suggest that partners of both isoforms have bigger and smaller domains respectively. However, this is not the case, because the number of domains is proportional to the number of amino acids in domains of each 14-3-3 paralog network (R^2^ = 0.9777, Fig. 3C). As shown in figure 3D, a big proportion of proteins from 14-3-3 full network are composed of a few domains (one or two), and the frequency of partners with more than two domains is similar between the seven isoforms. Three isoforms (gamma, theta and epsilon) have more partners with two domains than with one domain (Fig. 3D), in contrast to other 14-3-3 paralogs, including zeta and sigma, that have more partners with one domain. This suggests that the higher intrinsical disorder content of sigma’s partners must be allocated in their N or C terminals.

### The Partners of the Specific 14-3-3 Isoform’s Networks are Enriched in Different Domains

**Figure 4:**
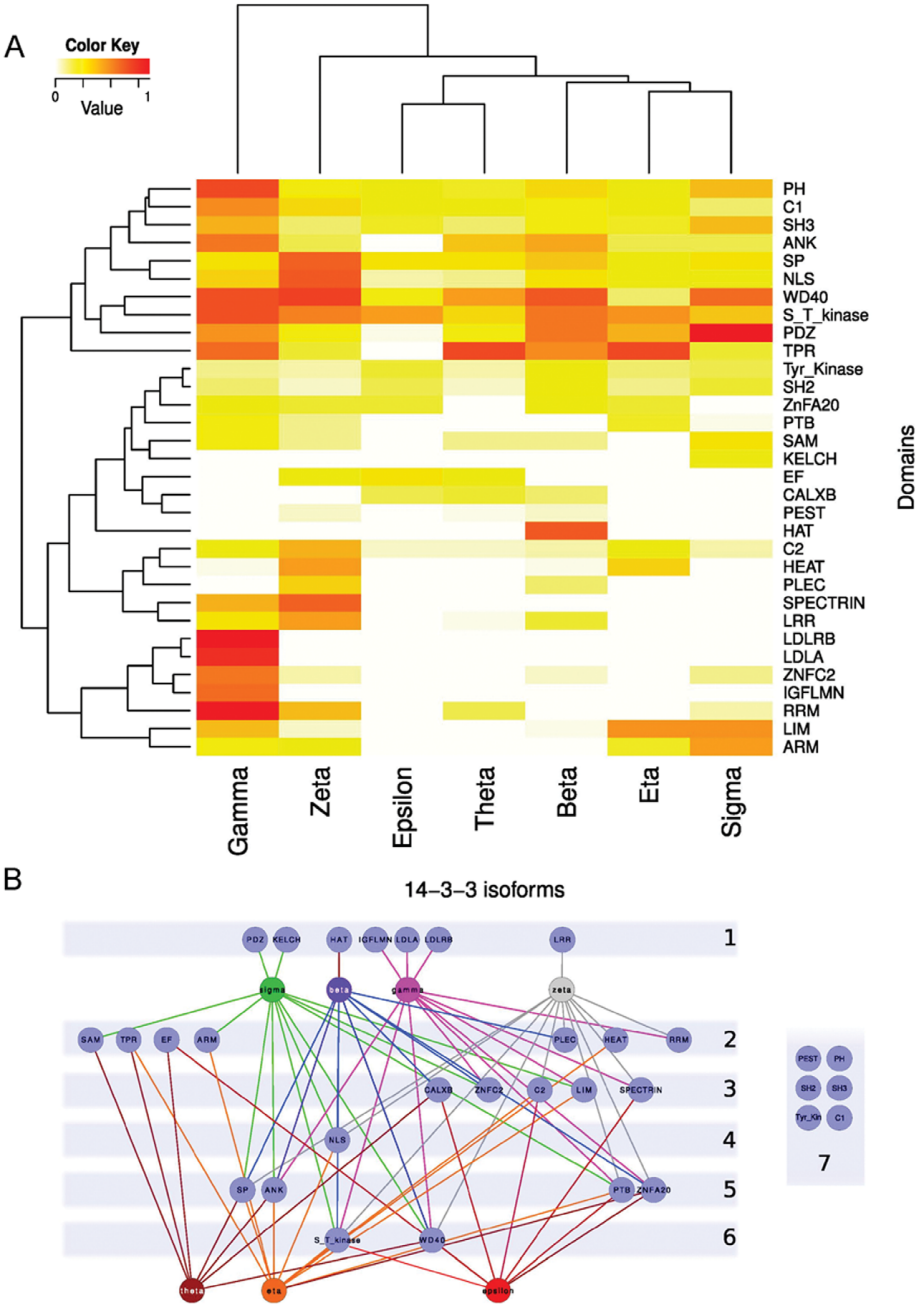
Domain composition of 14-3-3 isoforms’ partners. A) Heat map of the relative frequencies of protein domains within the seven 14-3-3 paralogs networks. The top 10 most abundant domains of each network were compared for all the networks. The color key represents the relative frequency, from white (lower value) to red (higher value). Fisher exact test comparison was performed (see text for details). B) Network representation of domains (light-blue nodes) interactions with 14-3-3 isoforms. Statistically significant over representation or absence of interaction is indicated by colored lines connecting each isoform with a domain node, except for the group of six domains that interact with all the isoforms, which were drawn apart for clarity. The numbers on the right correspond to the number of 14-3-3 isoforms interacting with each domain.

Figure 4 shows a heat map comparing the seven 14-3-3 paralogs networks frequencies of the top 10 more represented domains in the 14-3-3 partners (excluding the CC and TM domains, see Methods). Certain domains are over-represented in partners of one isoform but almost absent in others. That is the case for the low density lipoprotein receptor LDLR (A and B) domains. These are exclusive to the gamma network (6.43 e^−08^≤*p*≤0.00037 and 1.07 e^−08^≤*p*≤0.00023, for A and B respectively, Fisher exact test). The RNA recognition motif (RRM) domain is more frequent in partners of gamma isoform, over 5 of 6 other isoforms (9.34 e^−08^≤*p*≤0.0364, Fisher exact test). The HAT domain (half-A-TPR motif) is more represented in partners of beta over all other isoforms (8.42 e^−12^≤*p*≤5.13 e^−05^, Fisher exact test). Partners of sigma contain more (PSD-95/Discs- large/ZO-1) PDZ domains than all the other isoforms (3.25 e^−07^≤*p*≤0.00904, Fisher exact test); this domain generally binds to C-terminal motifs and in certain cases also recognizes specific phospholipids. Also, the commonly actin tail-associated domain KELCH is more represented in sigma network than those of the others isoforms (2.09 e^−05^≤*p*≤0.0129 Fisher exact test). LIM, a Cys and His rich domain that mediates PPI, is significantly more frequent in sigma over 4 other isoforms networks (2.47 e^−07^≤*p*≤0.00155, Fisher exact test). The TPR domain (probably the ancient domain from which the 14-3-3 proteins evolved) is more present in partners of theta isoform than in all others isoforms with exception of eta (2.53 e^−07^≤*p*≤0.0246, Fisher exact test). The calcium-binding domain EF is over represented in epsilon’s network (1.84 e^−07^≤*p*≤0.00498, Fisher exact test) compared with 5 of 6 isoforms networks. Finally, the LRR (Leu rich repeat) is specific of zeta network (7.24 e^−05^≤*p*≤0.0488, Fisher exact test), whereas the NLS is significantly over represented in zeta’s partners over gamma, theta and epsilon partners (0.0031≤*p*≤0.0219, Fisher exact test). This clearly shows that not all the domains are frequent in all isoforms networks; indeed, the heat map graph displays clearly different patterns of domain frequencies for each compared network, suggesting the involvement of specific 14-3-3 isoforms in different cellular pathways.

The figure 4B shows a network of the 14-3-3 isoforms and the domains more frequently represented in their interaction partners. To build this network, we constructed a matrix using *p* values (from Fisher exact test) and a cutoff of *p*≤0.01 to consider the significant presence or absence of each domain in each specific isoform network. Four isoforms (sigma, beta, gamma and zeta) have statistically differential representation of specific domains in their networks. The number of domains shared by 2 or 3 networks is greater than the domains shared by 4, 5 or 6 isoforms, evidencing again more isoform differences than similarities. Even more, there are no two domains shared by the same combination of two or three isoforms networks, making each network particular and intertwining the different 14-3-3 paralogs signaling. Because of the content of disordered regions and the postulated function of 14-3-3 proteins as molecular chaperones, we expected a high number of PEST domains in their partners. Our analysis revealed an unexpectedly low frequency of these domains, although present in all isoforms networks (Fig. 4B) with no significant differences (p>0.05, Fisher exact test).

Comparing the diversity of domains present in partners of each isoform, zeta and gamma have the most diverse number of domains followed by beta, sigma, theta, eta and epsilon (a complete list is in Table S4). A comparison of this list with the figure 3A shows that the domain diversity is not proportional to the number of total domains or amino acids in 14-3-3 partners.

Jin and co-workers developed a new algorithm to assign proteins to groups with related domain composition and functional properties, using multiple eukaryotic proteomes [35]. They defined social and isolated domains as those that are found in proteins with multiple domains and those that are found in single domain proteins and never linked to other domain types, respectively. In a hierarchical tree, social domains are found within the first 1080 clades, and isolated domains (∼1081 to 1250) branch directly from the root. The domain club was a concept introduced by Pawson [35] to define evolutionary patterns of domains that result in clustering of proteins based on their domain compositions. A group of domains that are regularly found in association is defined as a club. In a proteome wide analysis, Dr Pawson’s group found ∼1100 clubs and 200 isolated domains (those with no observable frequency of association). We clustered each 14-3-3 paralog’s client into a respective clade (from 1 to 1250, Fig. S1). We observed a strong representation in the clades which contain the S_T_kinase domain (#172 (S_T_Kinase/UBA) and #566 (S_T_kinase/FHA or DCK)) and in the clade including the TPR/HAT domains (club #224). The isoform zeta is the only one with a representation in the region of isolated domain. These domains are highly conserved in sequence and functions, and generally mediate central cellular functions. They cannot tolerate any linkage to other domain types, because this could prove deleterious to their core activity [35]. This is in agreement with the characteristic of zeta’s network to have high frequency of single domain proteins.

### The Partners in the Specific 14-3-3 Isoform’s Networks have Different Percentage of Phosphorylated Amino Acids

**Figure 5:**
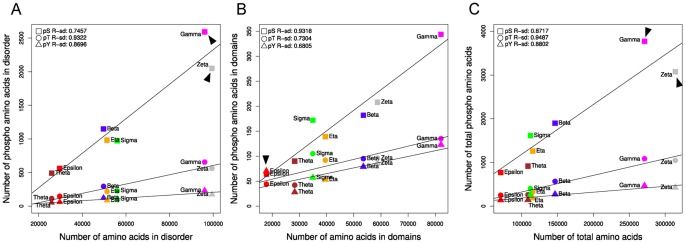
Relative abundance of phospho-amino acids in disordered, domains or total regions of 14-3-3 isoforms partners.

**Figure 6:**
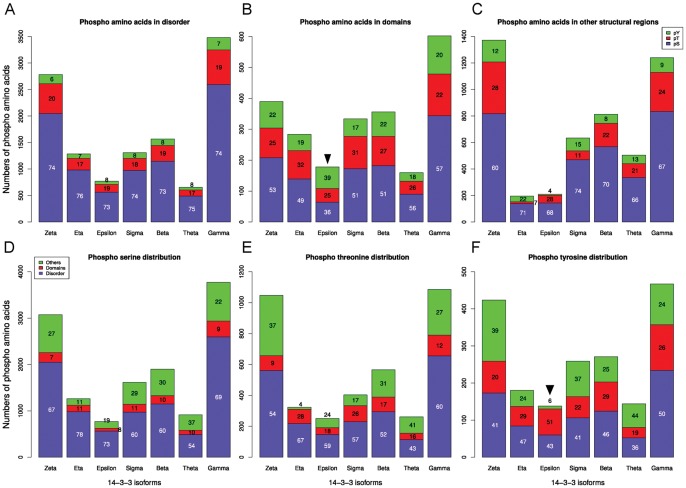
Numbers and percentages of phospho-amino acids in disordered, domains and total regions of 14-3-3 partners.

**Figure 7:**
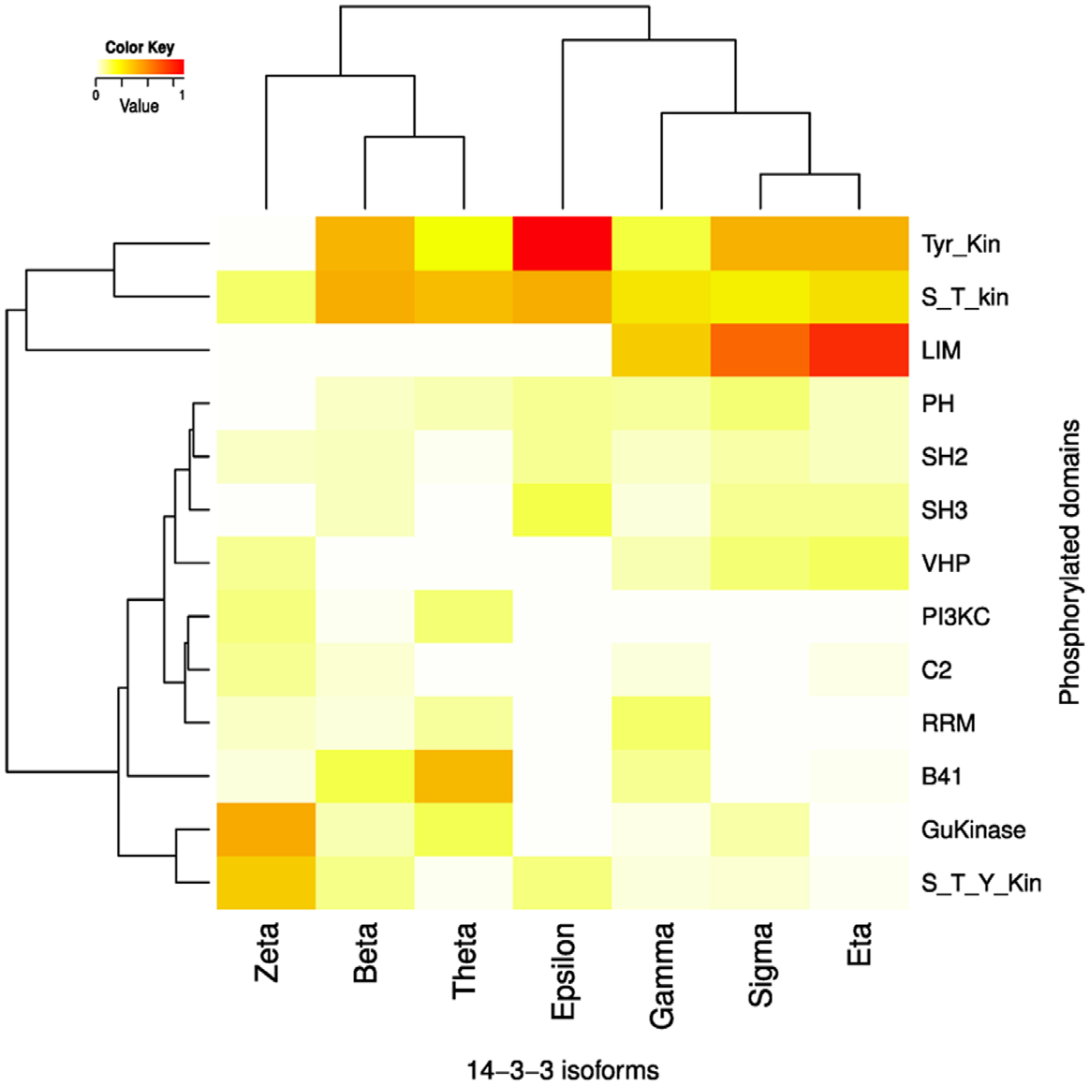
Phospho-domain composition of 14-3-3 isoforms’ partners. Heat map of the relative frequencies of protein phospho-domains within the seven 14-3-3 paralogs networks. The top 5 most abundant phospho-domains of each network were compared for all the networks. The color key represents the relative frequency, from white (lower value) to red (higher value). Fisher exact test comparison was performed (see text for details).

In order to find out about the participation of each 14-3-3 isoform in different signaling pathways we started analyzing the PTMs of each partner at high resolution (residue numbers and surrounding sequences). For each isoform, we investigated the relative abundance of phosphorylation sites of serine, threonine and tyrosine residues in the disordered regions, domains and total proteins. The number of each phospho amino acid is proportional to the number of residues in disordered regions (Fig. 5A). However, this changes significantly in domains of each 14-3-3 isoform network (Fig. 5B). In the case of the pTyr (lowest R^2^, Fig. 5B3), epsilon and theta isoforms are clearly the most dispersed ones. Partners of epsilon and theta contain more and less pTyr, respectively, compared with the other 5 isoforms. The absolute numbers and percentage of each phospho amino acid in three structural classifications (disordered, domains and other regions) is shown in figure 6. When disordered regions are compared, all the isoforms networks have almost the same percentage of each phospho amino acid. The pSer represents the majority (∼75%) of the phospho amino acids, followed by the pThr (∼20%) and the lowest proportion corresponds to the pTyr (∼8%). In domains, the values for each phospho amino acid change proportionally in 6 of the 7 isoforms networks. The pSer still represents the majority (∼53%), followed by pThr (∼25%), and then pTyr (∼20%). However, in epsilon partners, the percentages of pSer and pTyr in domains are inverted (Fig. 6B). While in all other isoforms networks serine is the most phosphorylated amino acid in domains, the value dropped to 36% in epsilon partners whereas pTyr raised to 39%, almost the double value compared to the ∼20% in partners of all the other isoforms. This difference is statistically significant compared to zeta, gamma, theta and beta isoforms (3.5e^−07^≤*p*≤1.3e^−03^, Fisher exact test). This suggests that 14-3-3 epsilon could be involved in growth factor receptor signaling pathway, together with Tyr_kinases, SH2 (that binds to specific pTyr-containing peptides), and SH3 domains (the SRH Homology 3 domain, that increases the substrate specify of some Tyr_kinases). Studying in detail the composition of phosphorylated domains in each isoform network (Fig. 7), we found that the phosphorylated Tyr_kinase domain is significantly more frequent in partners of the epsilon isoform compared with all the other isoforms (2.2 e^−6^<*p*≤2.20 e^−5^, Fisher exact test). Also, the phosphorylated domain SH3 is significantly more represented in epsilon compared to zeta, beta, gamma and theta networks (5.74 e^−06^≤*p*≤0.044, Fisher exact test). However, we didn’t find significant differences in the modified SH2 domain (the results are summarized in figure 7).

We then evaluated the relative involvement of the kinase groups [44] across the isoforms networks (Fig. S2). We observed that the majority of kinase groups have a comparable number of substrates across isoforms and didn’t generate a clear differential pattern between isoforms; however, some groups cluster together and differentiate from the others. The GMGC and AGC groups form a cluster and have the greatest number of substrates across 14-3-3 isoforms networks. CAMK and TK cluster together and differentiate from STE, TKL, atypical and CK1 groups with the smaller number of substrates in the 14-3-3 network (Fig. S2).

### Partners of 14-3-3 Zeta are More Acetylated in Lys Residues

**Figure 8:**
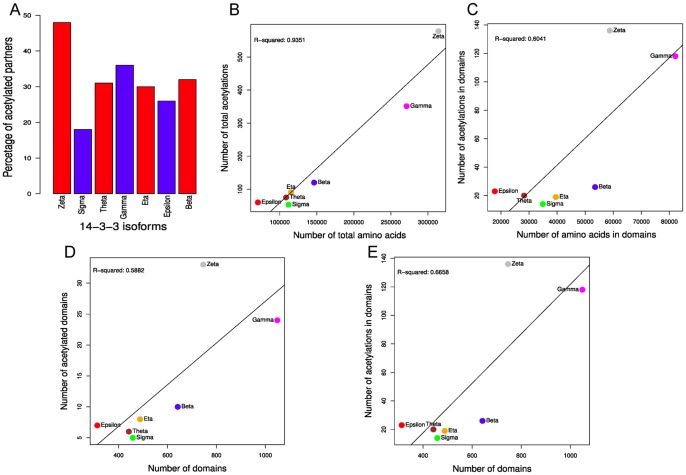
Relative abundance of acetylated partners and number of acetylated Lysines in 14-3-3 isoforms networks.

The lower content of disorder in the clients that interact with the zeta isoform suggests that besides phosphorylation, a PTM postulated as more likely to occur in ordered regions could be over represented in this group of proteins. We analyzed 10 different PTMs from the HPRD and found that the number of acetylated zeta’s partners in Lys residues is significantly higher than those of all other 14-3-3 isoforms (Fig. 8A, 1.65 e^−10^≤*p*≤0.0024, Fisher exact test. See Table S5 for the complete list of *p*-values). Although the number of acetylated Lys is proportional to the number of amino acids along the isoforms networks (Fig. 8B, R ^2^ = 0.9351), the fraction of this modification that occurs in domains is specifically higher in zeta network. The number of acetylated amino acids in domains is not proportional to the number of amino acids in domains (Fig. 8C) or number of domains (Fig. 8E) of each isoform network. Similarly, the number of acetylated domains is not proportional to the number of domains (Fig. 8D).We observed a larger percentage of acetylated zeta’s partners, with more modified Lys in domains than all others isoforms (Fig. 8). It has been reported that Lys acetylation is prone to occur in ordered regions [29], however in partners of the 14-3-3 full network, the number of acetylations occurring in domains is comparable to the number of the same modifications that happen in disordered regions or in non-classified regions (see Fig. S3). This is markedly different to Ser and Thr phosphorylation, which mainly occur in disordered regions, and Tyr phosphorylation, which fraction in domains is significantly increased. Besides the regulation of protein stability and interactions, acetylation could regulate the nucleo-cytoplasm shuttling. The analysis of subcellular localization of the 48% of zeta partners that are acetylated shows that 42% of them are mainly nuclear, containing 60% of all the NLS present in partners of 14-3-3 zeta (*p* = 1.288 e^−06^, Fisher exact test).

**Figure 9:**
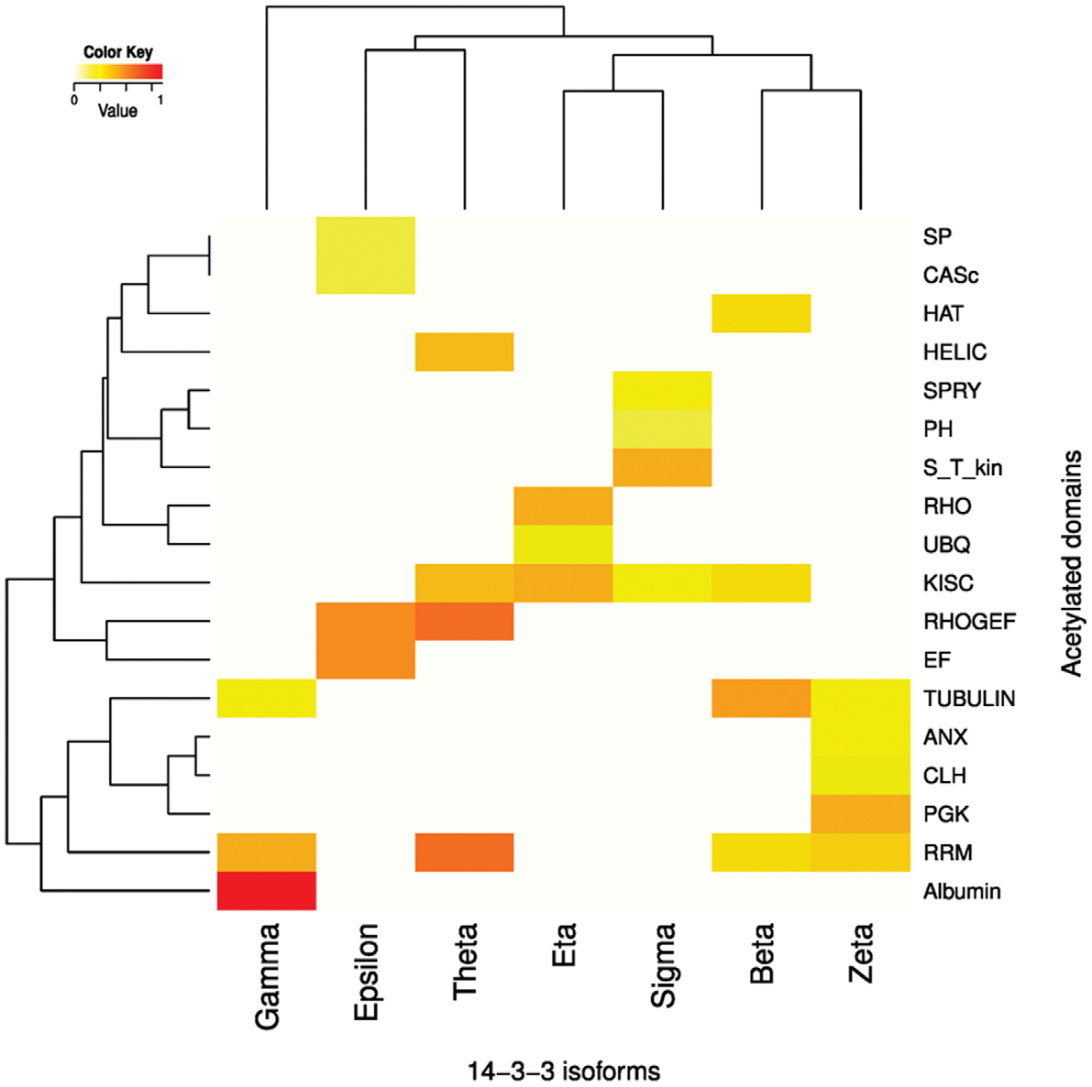
Acetylated domain composition of 14-3-3 isoforms’ partners. Heat map of the relative frequencies of protein acetylated domains within the seven 14-3-3 paralogs networks. The top 5 most abundant acetylated domains of each network were compared for all the networks. The color key represents the relative frequency, from white (lower value) to red (higher value). Fisher exact test comparison was performed (see text for details).

Figure 9 shows the frequency of the top 5 acetylated domains of each isoform network (excluding the CC domains, see Methods section). It is to be noted that each network has a different pattern of acetylated domains, most of which are exclusively acetylated in one of the networks. The RNA-binding motif RRM is one of the over-represented in ac-Lys containing domains. Others domains, previously identified in acetylome studies, and associated with nuclear functions as helicases, PWWP, PHD finger or bromodomains were not frequent in the 14-3-3’s interactome.

The EF domain, over-represented in epsilon and theta networks, is acetylated exclusively in partners of epsilon (Fisher exact test). An unexpected result was that S_T_kinase domains are exclusively acetylated in partners of 14-3-3 sigma (Fisher exact test), raising the question if this modification inhibit or change the S_T_kinase activity.

### Crosstalk between Phosphorylation and Acetylation

**Figure 10:**
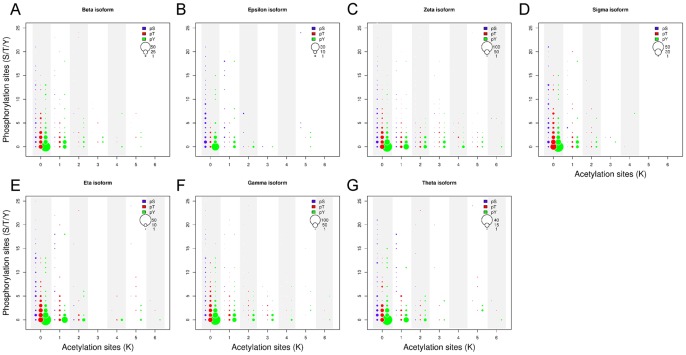
Bubble plot of number of phosphorylation *vs* acetylation sites within 14-3-3 partners. The size of each circle is proportional to the number of partners with that combination of phosphorylated and acetylated residues. The partners corresponding to the different 14-3-3 paralogs where plotted in separated graphs (A to G).

We are interested on the study of the cross relation between lysine acetylation and serine, threonine or tyrosine phosphorylation in partners of 14-3-3 paralogs. To analyze this, the number of proteins containing a pair of both PTM types (phosphoserine/acetyl lysine, phosphothreonine/acetyl lysine or phosphotyrosine/acetyl lysine) contained on each 14-3-3 network was graphed in a bubble plot (Fig. 10). A significant fraction of the proteins are modified by more than one PTM. Lysine acetylation and tyrosine phosphorylation sites appeared tightly coupled: the majority of tyrosine phosphorylated proteins were also found lysine-acetylated, showing a correlation of these two posttranslational modifications (*p*>0.01 in 5 of 7 isoforms, Fisher’s exact test). However, phosphorylation on serine and threonine appeared as independent events from lysine acetylation (*p*<1.51 e^−13^ and *p*<1.33 e^−08^ respectively, Fisher’s exact test. See Table S6 for a complete list of *p*-values).

We box plotted the numbers of modifications in serines, threonines, tyrosines and lysines of each partner of 14-3-3 discriminated by structural features (disordered regions and domains) and total amino acids (Fig. S3). While on average <10% of all serines, threonines, tyrosines and lysines are modified [25], some proteins exhibit an unusually high level of modifications. The phosphorylation in serines residues is significantly higher in all isoforms partners in disordered regions. However, in structured regions as domains, the proportions of acetylation of lysines and phosphorylation of tyrosines and serines are similar (Fig. S3).

### 14-3-3 Isoforms Networks Differ in Gene Ontologies Enrichment

**Figure 11:**
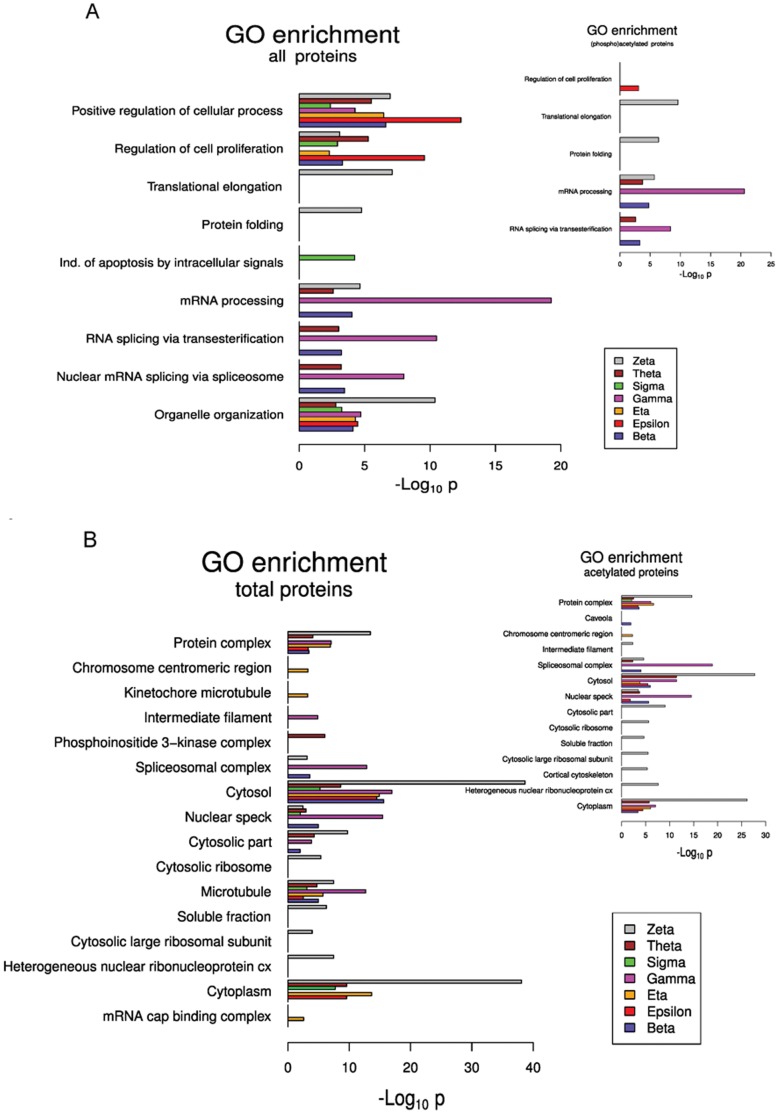
Gene ontology (GO) enrichment within each 14-3-3 paralog networks. A) GO: Biological Process, B) GO: Cellular component. The analysis of the acetylated networks is included as an inset.

We used the BiNGO plugin [36] from Cytoskape [37] to analyze whether the protein interaction networks of the different 14-3-3 isoforms are statistically enriched in partners associated with particular biological processes and/or subcellular localizations. Among the 7 mammalian isoforms, 4 (gamma, zeta, epsilon and sigma) interaction networks are significantly more enriched in specific biological processes than the others (Fig. 11A). In accordance to its enrichment in RRM motif, gamma network is specifically enriched in RNA processing, particularly in RNA splicing *via* transesterification reactions and *via* spliceosoma. This is not the only 14-3-3 isoform network enriched in these biological processes, but the others (zeta, theta and beta) are between two and seven orders of magnitude lower on the significance scale (-log *p* value). Zeta is the only isoform whose partners are enriched in translational elongation and protein folding. It is also enriched on organelle organization, with *p* values more than two orders of magnitude lower than all the other isoforms. Similarly, epsilon network is enriched in positive regulation of cellular processes and regulation of cell proliferation. Although epsilon, eta, theta and zeta interaction networks are enriched in induction of apoptosis, sigma network is the only one enriched on induction of apoptosis by intracellular signals. Other biological processes are more represented in two or more isoforms networks (i.e. cellular component assembly, zeta and gamma); to simplify, these were not included on the graph. The analysis of biological processes within acetylated partners showed a subset of these same enrichments (Fig. 11A and inset).

Cellular component analysis also showed significant GOs enrichment of some isoform networks. In this case, five of the seven 14-3-3 paralogs (zeta, gamma, eta, beta and theta) networks are more enriched in specific GOs (Fig. 11B). In accordance with their biological processes enrichments, zeta network is more enriched in protein complex, cytoplasm and cytosol and is the only network enriched in cytosolic ribosome, cytosolic large ribosomal subunit and heterogeneous nuclear ribonucleoprotein complex. Gamma network is enriched in spliceosomal complex, nuclear speck and microtubules. Eta network is the only one enriched in two related cellular components, chromosome centromeric region and kinetochore microtubules; and theta in phosphoinositide 3-kinase complex (Fig. 11B). In this case, acetylated networks also showed enrichment in a subset of these same GOs, except that a new GO (Caveola) is enriched in beta acetylated network (Fig. 11B inset). Interestingly, comparing cellular component enrichment on total or acetylated networks, 4/16 (25%) of the differentially enriched GOs were exclusively enriched within zeta network, and this tendency was even higher comparing the acetylated networks, in which 8/14 (57%) of GOs were zeta network specific. Also, it is to be noted that considering both, biological processes and cellular component, all isoforms are differentially enriched in at least one GO, suggesting again certain degree of specialization.

## Discussion

We performed a study that comparatively analyses several PTM types in networks of the 14-3-3 paralogs family. All cellular functions of 14-3-3 proteins are not fully elucidated yet, but as a rule, these proteins act by binding to phospho-protein ligands, thus regulating their activity [6]. Some functions can be carried out indistinctly by any isoform; however, a growing number of functions have been demonstrated to be isoform-specific. This opens the hypothesis that subfunctionalization could be a possible explanation of how a family with 7 paralogs was evolutionary retained. This has been identified as a non-adaptive mechanism for the retention of duplicate genes in small-population species, like mammals or plants [45], [46].

The list of non-redundant human proteins interacting with each 14-3-3 paralog, kinases and kinase substrates was recovered from the Protein Interaction Network Analysis (PINA) platform, which integrates PPI data from six different databases (IntAct, MINT, BioGRID, DIP, HPRD and MIPSMPact). The dataset was also manually revised and curated using the information of non-interacting proteins from HPRD (the file was downloaded from http://www.hprd.org/download).

High throughput data could include some proteins that do not bind directly to 14-3-3s, but rather interact with other phospho-proteins that in turn interact with 14-3-3s. In this hypothetical case, or if future experiments find more isoforms involved in some previously characterized specific interactions, our conclusions will remain unaltered because they are based on a systems biology approach. The literature about 14-3-3 is full of low-throughput experiments, some of them with overlapping information with the high-throughput experiments. Most of these studies contain detailed information about the physical interactions between 14-3-3 and their clients. Our dataset includes information from a well-balanced literature of both types of studies, the high- and low-throughput experiments.

The complete network information used in this work is summarized in Table S1, (.csv file). This data can be easily uploaded on the freely available Cytoscape program to directly obtain an interactive visualization of the network. Some isoforms could form heterodimers, however, this phenomenon does not affect the interpretation of our analysis, which is a comparison among the isoforms networks. As there is some degree of overlapping between the networks, it can be reasonable to suppose that a heterodimer would interact with a partner included in the networks of both monomers that form the heterodimer. It is also possible that the partner binds only to one of the monomers of the heterodimer, in such case it could be contained only in one of both networks.

A few non-phosphorylated binding sites have been reported on some 14-3-3 interaction partners. To our knowledge, those are as few as five cases, only three of which are included on the human proteome. This represents a very small fraction (0.6%) of the phosphorylation dependent interactions with 14-3-3 that we analyzed. For these reasons, we consider those as exceptions more than a significant phenomenon and were excluded from our analysis.

Using a manually curated network of 14-3-3 proteins in human cells, we searched for signs of subfunctionalization from the common function of scaffold protein. First, we demonstrated that directed networks of partners of each 14-3-3 paralog are conformed by different motifs. One motif family found was the feedforward loop (#7). It appears in hundreds of gene systems in *E. coli* and *S. cereviceae*, as well as in other organisms [40]. This motif was originally described as three genes: a regulator X, which regulates Y, and the gene Z, which is regulated by both X and Y, and is one of the most stable motifs [41]. In a signal transduction scenario, this motif is also highly represented and can be interpreted as the protein scaffold activity. 14-3-3 proteins may act as scaffolds, as the dimers have the potential to bind simultaneously to two different proteins. This motif is over represented in networks of eta, gamma and zeta 14-3-3 isoforms. Another motif that conform stability to the networks is the linear sequential regulation motif (#3), which is over represented in eta, epsilon and theta 14-3-3 paralogs.

The analysis of protein disorder allowed us to understand that partners of each 14-3-3 paralog have different properties, because disordered regions are segments of a protein that does not completely fold and remains flexible and disordered [47]. Distinct PTMs have different propensities to occur in disordered or order regions. In general, phosphorylation is highly frequent in disordered regions [48], whereas acetylation of Lys is 75% more frequent in order ones (helix and sheets) [27]. Even though phosphorylation and acetylation appear to be co-evolutionary conserved as previously shown in several studies [24], [25], [27], [29], [49], our results show that at least in 14-3-3-linked phosphorylation, acetylation is associated with tyrosine phosphorylation and not with serine or threonine phosphorylation.

In the 14-3-3 interactome it shows up a clear difference in the domain preference of each 14-3-3 paralog, both in individual domains and in groups. The domains S_T_Kinase (alone or in the club #172 (S_T_Kinase/UBA) and #566 (S_T_kinase/FHA or DCK)) and TPR (or club #224 (TPR/HAT)) are clearly present in most of the isoforms networks. However, there are specialized domains present exclusively in one network. Also, there are differences in social and isolated domains; 14-3-3 zeta is the only isoform with a high representation of isolated domains (Fig. S1). This shows that the 14-3-3 protein family is strongly involved in phosphorylation and PPI signaling, but also that there are specializations through domains that are present in one or two isoforms networks only. This is interesting in the scenario of eukaryotic domain evolution, where a small number of domains occur in many proteins, and most of them are found only in a few proteins [50]. One of the most interesting examples within the 14-3-3 family is the epsilon isoform. Globally, phosphorylation takes place mainly on serine residues (86.4%), followed by threonine residues (11.8%) and tyrosine residues (1.8%) [51], [52]. However, epsilon network is specifically enriched in phosphotyrosines inside domains and phosphorylated domains as Tyr_Kinase and SH3. This suggests that the epsilon network could be involved in the regulation of the growth factor receptor signaling pathway, which transduces key extracellular signals triggering cellular events and physiological processes. This signaling process is quite complex; upon ligand binding, the receptor undergoes a series of dimerization and autophosphorylations at tyrosines residues. These phosphorylated tyrosines consequently become binding sites for a variety of intracellular SH2 domain–containing proteins such as phospholipase, phosphatidylinositol 3-kinase p85 subunit, Ras GTPase–activating protein, etc. Some act as binding sites for SH2-SH3 adaptor proteins, activating a diverse array of signaling pathways including the Ras, PLCg, and PI3K pathway. The 14-3-3 isoform epsilon could be the link for the communication between these various signaling pathways after the growth factor receptor pathway activation.

Besides the regulation of protein stability, activity and PPI, acetylation has especial influence on nuclear import and export of proteins [53]. Most proteins that shuttle between the nucleus and the cytoplasm are acetylated by the histone acetyltransferase activity of the transcriptional co-activator proteins p300/CBP, whose targets include HNF-4, CIITA, PCNA, SRY, cAbl, CtBP2, p53, PAP, and β-catenin. By means of different mechanisms like modification of the interaction with a binding partner or with nuclear import/export factors, acetylation can enhance localization in the cytoplasm for some proteins, whereas for others it can favor a nuclear localization [53]. Until now however, no general rules for the localization of the acetylated subpopulation of proteins has been postulated. Interestingly, the NLS of HNF-4 has to be acetylated in order to be retained in the nucleus. Similarly, in 14-3-3 zeta network, a statistically significant proportion of the acetylated partners is nuclear and contains NLS. This is consistent with the accumulation in the perinuclear region and nucleus of this isoform as determined by immunofluorescence [17] and the high proportion of nuclear partners of zeta (40%, Table S1).

Our study enabled us to find several differences between paralog members of the 14-3-3 protein family and points out their putative subfunctionalization. The preservative role of subfunctionalization in humans and other higher eukaryotes is the result of mild mutations likely to cause a differential expression-regulation-function in gene duplicates [46]. We postulate that mechanistically these mutations must have occurred outside the binding pocket formed by the strictly conserved triplet of amino acids (K49, R56 and R127) in paralogs of 14-3-3.

As MacKintosh defined [7], the 14-3-3-binding Ser/Thr phosphosite present in their partners is a conserved lynchpin, leaving the rest of the molecule free to evolve by fusion events, exon shuffling and domain insertions or deletions, followed by simple point mutations [50]. This can first determine paralogs specificity through a second binding pocket, similar to the one we found in the 14-3-3/AANAT complex [21], and secondly by paralogs subfunctionalization.

Our results imply previously unreported hidden differences of the 14-3-3 isoforms interaction networks. The phosphoproteome and lysine acetylome within each network revealed post-transcriptional regulation intertwining phosphorylation with lysine acetylation, especially evident in zeta interaction network. A global understanding of these phospho-acetylation networks will additionally contribute to predict what could occur when regulatory circuits become dysfunctional or are modified in response to external stimuli.

## Supporting Information

Figure S1 **Heat map (relative frequencies) of social and isolated domains clusters within the 14-3-3 paralogs networks.** The color key represents the relative frequency, from white (lower value) to red (higher value). Each 14-3-3 paralog’s client was assigned to a clade with related domain compositions and functional properties using the algorithm developed by the same authors.
(TIF)

Figure S2 **Heat map of the relative frequencies of kinases families within the seven 14-3-3 paralogs networks.** The color key represents the relative frequency, from white (lower value) to red (higher value).
(TIF)

Figure S3 **Number of modifications (box plot) for serines, threonines, tyrosines and lysines of 14-3-3 paralogs networks.** The data were discriminated by structural features (disordered regions, domains and total). The partners corresponding to the different 14-3-3 paralogs where plotted in separated graphs.
(TIF)

Figure S4 **Cytoscape graph (BiNGO) of the GO categories from Biological Process enrichment for 14-3-3 paralogs networks.** For each isoform, the maximum *p* value was settled to obtain a Cytoscape graph containing between 35 to 50 GOs. Yellow and orange nodes represent terms with significant enrichment, darker orange represents a higher significance; white nodes are terms with no significant enrichment. The size of each node is proportional to the number of nodes in the query set with that term. Small stars indicate branch-terminal significantly enriched GOs that are not directly related to kinases or phosphorylation. A) beta, B) epsilon, C) eta, D) gamma, E) sigma, F) theta, G) zeta.
(TIF)

Figure S5 **Same as Fig. S4. except that the acetylated sub-networks were analyzed.** A) eta, B) gamma, C) zeta. The 4 isoforms sub-networks that are not represented where not enriched in any specific GO.
(TIF)

Figure S6 **Cytoscape graph of the GO categories from Cellular Component enrichment for the 14-3-3 paralogs networks. Same as Fig. S4.** A) eta, B) eta acetylated, C) gamma, D) gamma acetylated, E) zeta, F) zeta acetylated.
(TIF)

Table S1 **14-3-3, kinase and kinase substrate full network in XML format.**
(CSV)

Table S2 **Jaccard indexes of the 14-3-3 isoforms networks.**
(PDF)

Table S3 **Wilcoxon rank sum and Kruskal-Wallis** ***p*** **-values from the comparisons between isoforms networks disorder (** **Fig. 2** **).**
(PDF)

Table S4 **Comparing the diversity of domains present in partners of each 14-3-3 isoform.** Zeta and gamma have the most diverse number of domains followed by beta, sigma, theta, eta and epsilon.
(PDF)

Table S5 **Fisher exact test (** ***p*** **-values) results of number of acetylated partners from each 14-3-3 paralog.**
(PDF)

Table S6 **Fisher exact test (** ***p*** **-values) results of number of each modified amino acid from 14-3-3 paralogs.**
(PDF)
