# Supplementary figures and images for: Human 14-3-3 Paralogs Differences Uncovered by Cross-Talk of Phosphorylation and Lysine Acetylation
Source: PLoS One. 2013 Feb 13;8(2):e55703. doi: 10.1371/journal.pone.0055703 (PMC3572099; doi:10.1371/journal.pone.0055703)

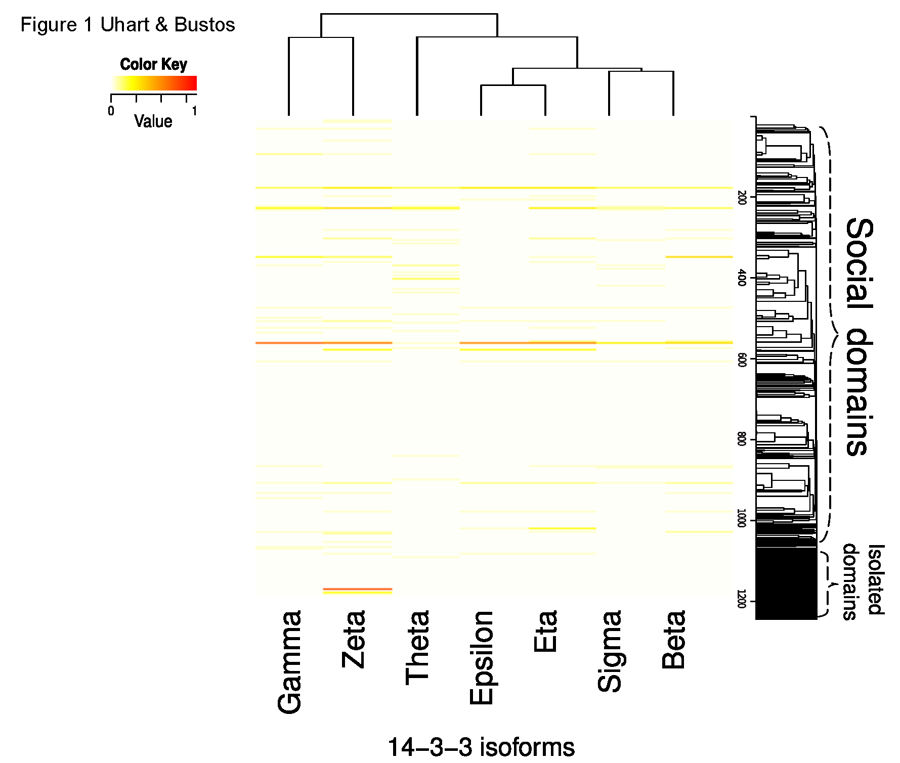

Supplement: Figure S1 — Heat map (relative frequencies) of social and isolated domains clusters within the 14-3-3 paralogs networks. The color key represents the relative frequency, from white (lower value) to red (higher value). Each 14-3-3 paralog’s client was assigned to a clade with related domain compositions and functional properties using the algorithm developed by the same authors. (TIF) [file pone.0055703.s001.tif]

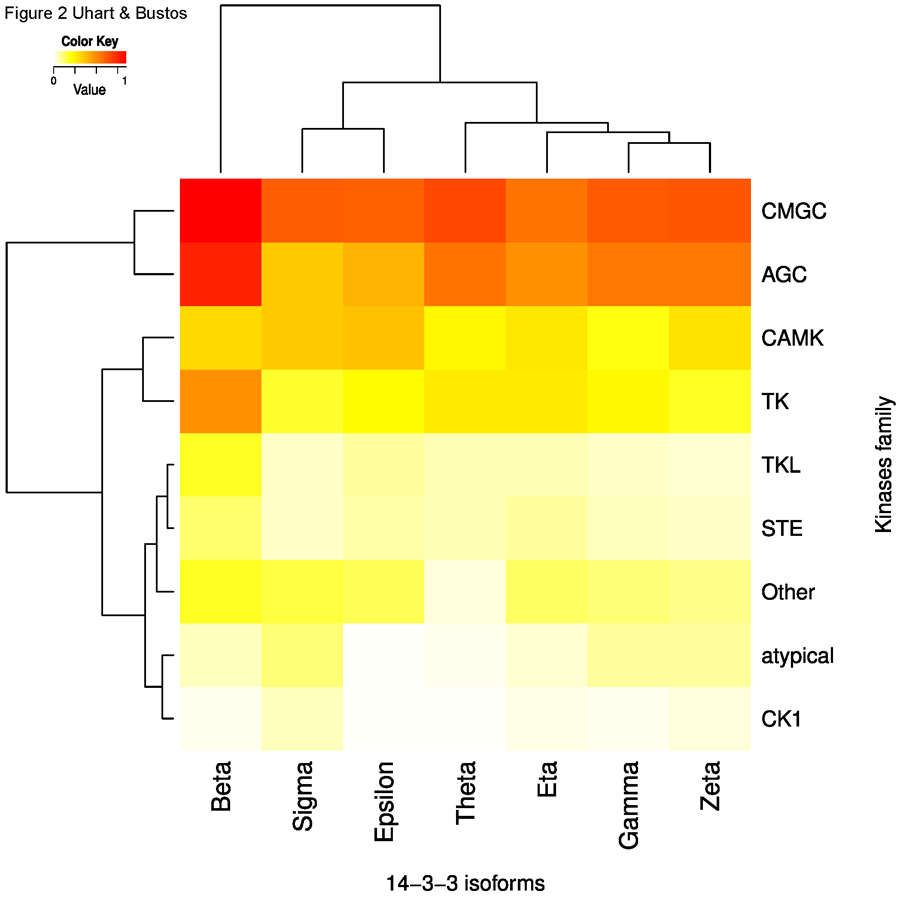

Supplement: Figure S2 — Heat map of the relative frequencies of kinases families within the seven 14-3-3 paralogs networks. The color key represents the relative frequency, from white (lower value) to red (higher value). (TIF) [file pone.0055703.s002.tif]

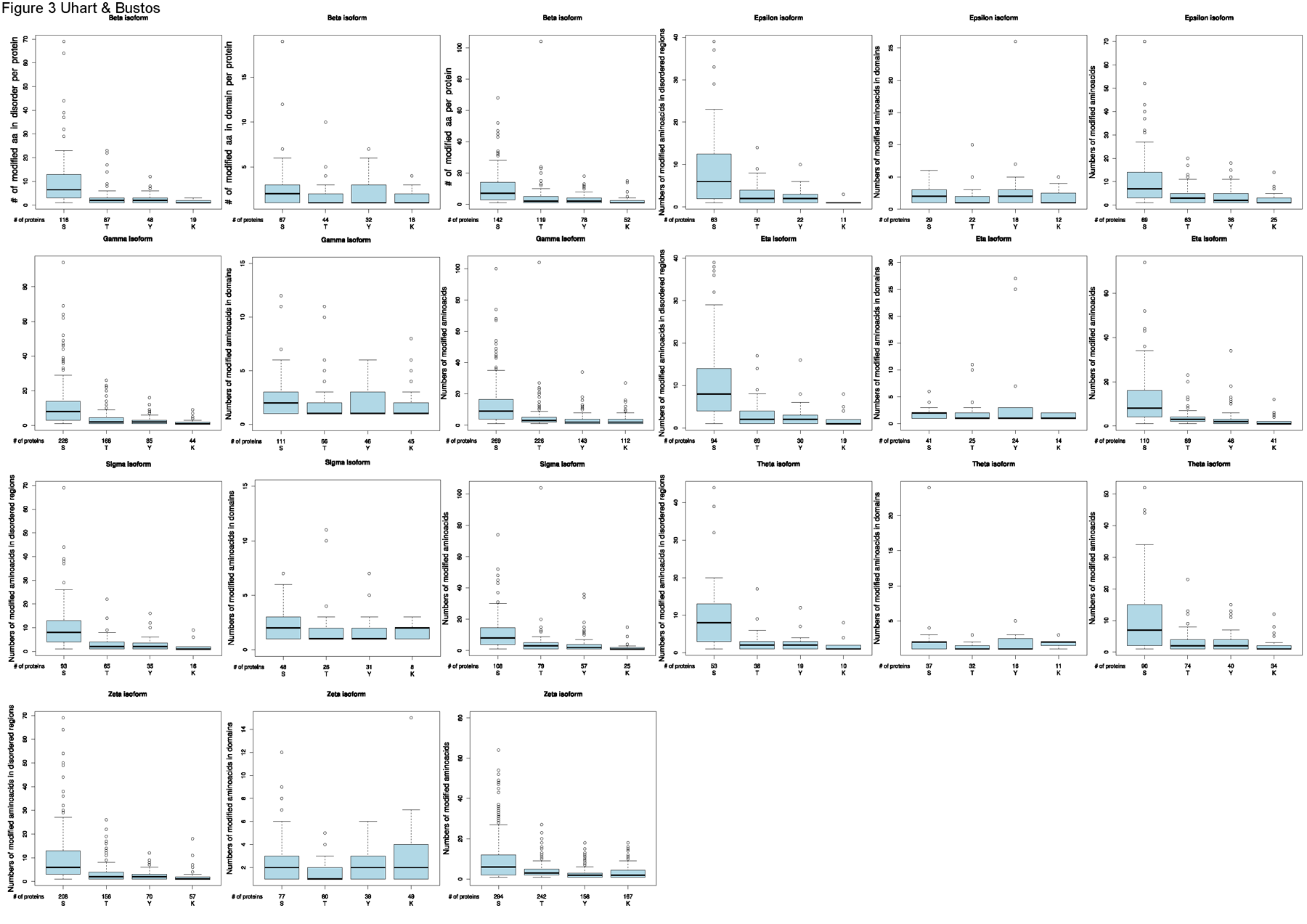

Supplement: Figure S3 — Number of modifications (box plot) for serines, threonines, tyrosines and lysines of 14-3-3 paralogs networks. The data were discriminated by structural features (disordered regions, domains and total). The partners corresponding to the different 14-3-3 paralogs where plotted in separated graphs. (TIF) [file pone.0055703.s003.tif]

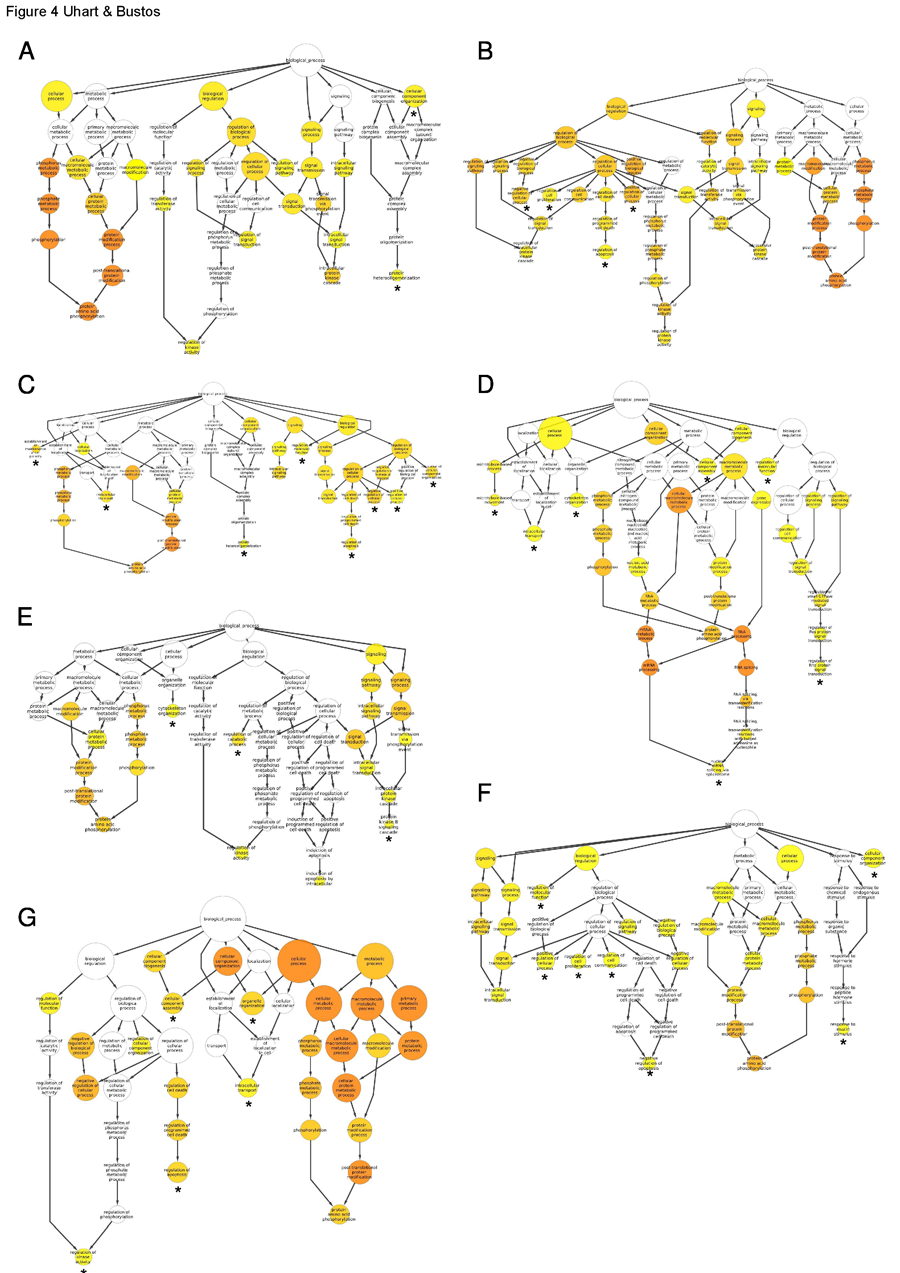

Supplement: Figure S4 — Cytoscape graph (BiNGO) of the GO categories from Biological Process enrichment for 14-3-3 paralogs networks. For each isoform, the maximum p value was settled to obtain a Cytoscape graph containing between 35 to 50 GOs. Yellow and orange nodes represent terms with significant enrichment, darker orange represents a higher significance; white nodes are terms with no significant enrichment. The size of each node is proportional to the number of nodes in the query set with that term. Small stars indicate branch-terminal significantly enriched GOs that are not directly related to kinases or phosphorylation. A) beta, B) epsilon, C) eta, D) gamma, E) sigma, F) theta, G) zeta. (TIF) [file pone.0055703.s004.tif]

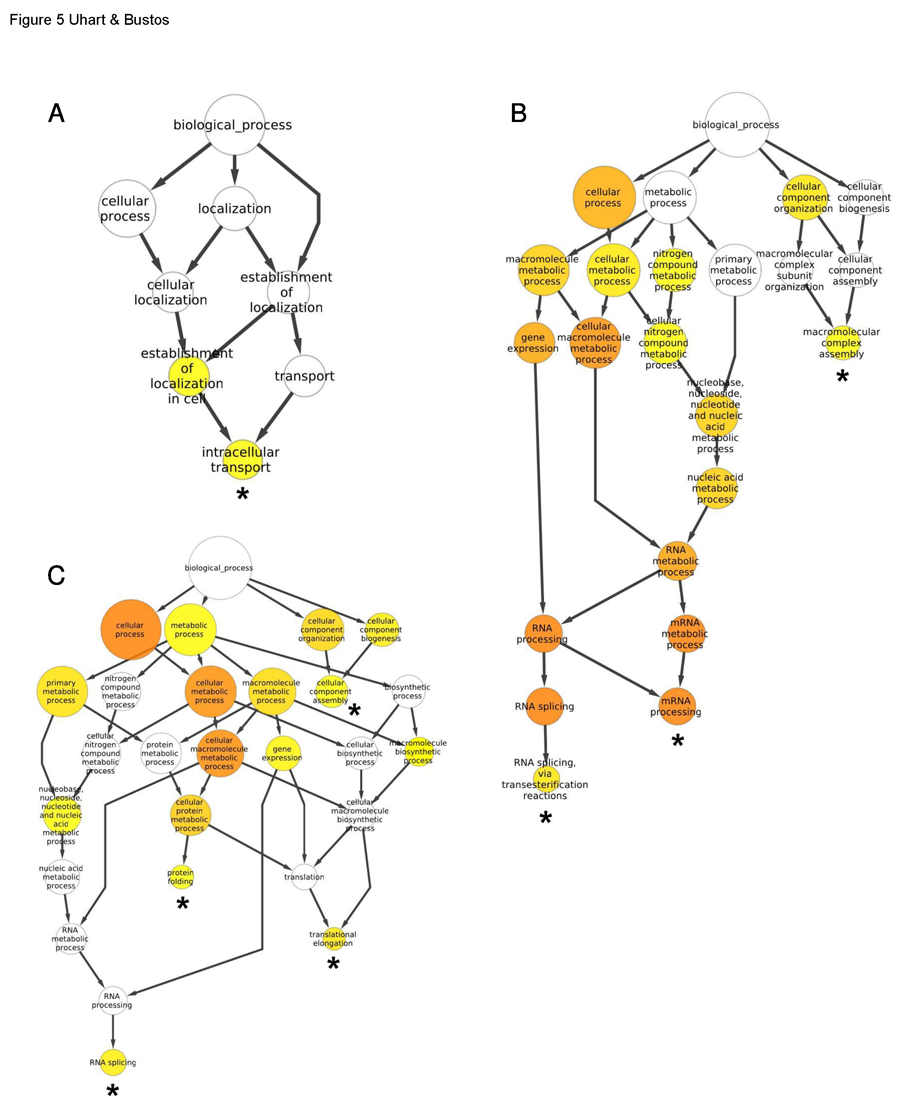

Supplement: Figure S5 — Same as Fig. S4. except that the acetylated sub-networks were analyzed. A) eta, B) gamma, C) zeta. The 4 isoforms sub-networks that are not represented where not enriched in any specific GO. (TIF) [file pone.0055703.s005.tif]

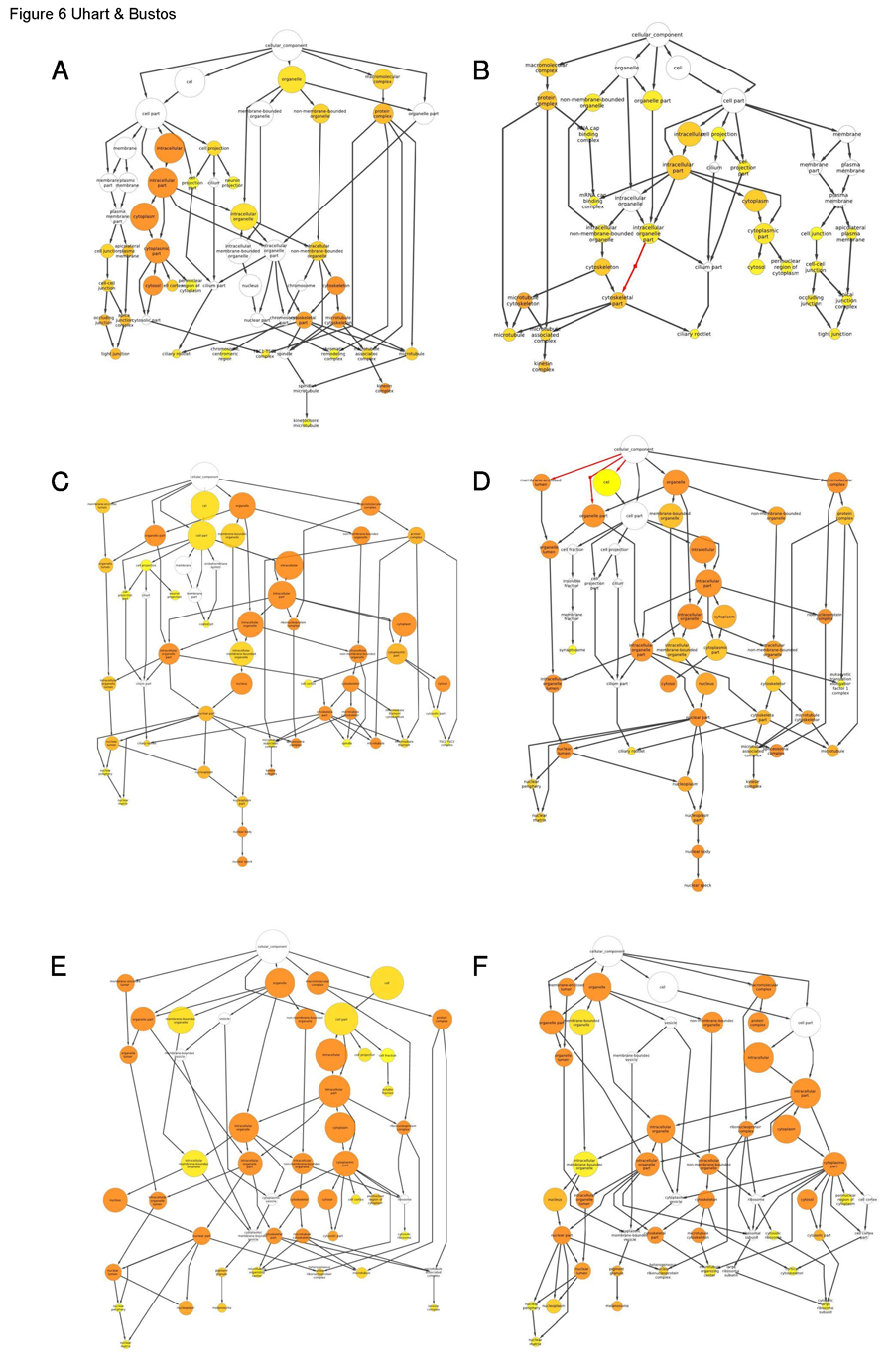

Supplement: Figure S6 — Cytoscape graph of the GO categories from Cellular Component enrichment for the 14-3-3 paralogs networks. Same as Fig. S4. A) eta, B) eta acetylated, C) gamma, D) gamma acetylated, E) zeta, F) zeta acetylated. (TIF) [file pone.0055703.s006.tif]
