# Supplementary material for: Human 14-3-3 Paralogs Differences Uncovered by Cross-Talk of Phosphorylation and Lysine Acetylation
Source: PLoS One. 2013 Feb 13;8(2):e55703. doi: 10.1371/journal.pone.0055703 (PMC3572099; doi:10.1371/journal.pone.0055703)
Supplement: Table S2 — Jaccard indexes of the 14-3-3 isoforms networks. (PDF) [file pone.0055703.s008.pdf]

Table 2: Comparison of each 14-3-3 isoform network by Jackard method

	Sigma	Eta	Theta	Gamma	Beta	Epsilon	Zeta
Sigma	-	0.149	0.133	0.151	0.170	0.123	0.105
Eta		-	0.216	0.203	0.196	0.209	0.168
Theta			-	0.173	0.273	0.178	0.143
Gamma				-	0.263	0.102	0.198
Beta					-	0.257	0.193
Epsilon						-	0.257
Zeta							-
