# Supplementary material for: Human 14-3-3 Paralogs Differences Uncovered by Cross-Talk of Phosphorylation and Lysine Acetylation
Source: PLoS One. 2013 Feb 13;8(2):e55703. doi: 10.1371/journal.pone.0055703 (PMC3572099; doi:10.1371/journal.pone.0055703)
Supplement: Table S3 — Wilcoxon rank sum and Kruskal-Wallis p -values from the comparisons between isoforms networks disorder ( Fig. 2 ). (PDF) [file pone.0055703.s009.pdf]

Table 3. Statistics *p*-values results from the comparisons between isoforms networks disorder content.

14-3-3 isoforms compared	*p*-value (Wilcoxon rank sum test with continuity correction)	$p < 0.05$ (*)
sigma vs epsilon	0.3501	
sigma vs eta	0.2767	
sigma vs gamma	0.0926	
sigma vs beta	0.004919	*
sigma vs theta	0.008575	*
sigma vs zeta	$5.863e^{-08}$	*
epsilon vs eta	0.4324	
epsilon vs gamma	0.2119	
epsilon vs beta	0.02994	*
epsilon vs theta	0.03828	*
epsilon vs zeta	$1.23e^{-05}$	*
eta vs gamma	0.2346	
eta vs beta	0.0262	*
eta vs theta	0.03737	*
eta vs zeta	$1.55e^{-06}$	*
gamma vs beta	0.08928	
gamma vs theta	0.1156	
gamma vs zeta	$1.101e^{-06}$	*
beta vs theta	0.4771	
beta vs zeta	0.0009975	*
theta vs zeta	0.002538	*

Comparison of all isoforms partners with Kruskal-Wallis test *p*-value = $2.044e^{-09}$
