# Supplementary material for: Human 14-3-3 Paralogs Differences Uncovered by Cross-Talk of Phosphorylation and Lysine Acetylation
Source: PLoS One. 2013 Feb 13;8(2):e55703. doi: 10.1371/journal.pone.0055703 (PMC3572099; doi:10.1371/journal.pone.0055703)
Supplement: Table S4 — Comparing the diversity of domains present in partners of each 14-3-3 isoform. Zeta and gamma have the most diverse number of domains followed by beta, sigma, theta, eta and epsilon. (PDF) [file pone.0055703.s010.pdf]

Table 4. Comparing the diversity of domains present in partners of each isoform

zeta		beta		epsilon		eta		gamma		sigma		theta	
domain	num. of occurrences	domain	num. of occurrences	domain	num. of occurrences	domain	num. of occurrences	domain	num. of occurrences	domain	num. of occurrences	domain	num. of occurrences
AAA	11	ANK	18	BH3	3	ANK	6	AAA	5	ANK	6	ANK	15
ACR	2	B41	11	C1	9	ARM	10	ABH	1	ARM	19	B41	7
ACTIN	1	BH3	5	C2	2	B41	3	ADF	1	B41	3	BH3	3
ADF	1	C1	11	CALXB	6	BH3	3	Albumin	3	BBOX	1	C1	8
ANK	6	C2	3	CASc	1	BRK	1	ANK	23	BCL	1	C2	2
ANX	8	CALXB	5	CC	69	BROMO	2	ARFGAP	3	BH3	3	CALPAIN_III	1
ARM	9	CC	117	CNMP	1	BTB	1	ARM	11	BRCT	1	CALXB	7
B41	5	CH	1	CUE	1	C1	8	B41	10	BTB	1	CBS	1
BAH	2	CNH	1	EF	12	C2	9	BBOX	1	C1	5	CC	96
BBC	1	COLL	1	ERM	1	CC	131	BH3	2	C2	3	CYSPC	1
BBOX	3	DEATH	1	FHA	1	CNMP	1	BTB	4	CC	80	DEATH	1
BROMO	1	DEXDc	3	FN3	3	CT	1	C1	21	CNH	1	DEXDc	2
BTB	1	DSH	1	FU	4	DEXDc	1	C2	8	ERM	1	DSH	1
C1	13	DSRM	1	HLH	1	FCH	1	CC	271	FU	3	EF	9
C2	17	EGF	1	HSF	1	FH	1	CH	8	FYVE	2	EGF	1
CAAX	1	ERM	1	IPT	3	FHA	3	CLH	7	GuKinase	4	ERM	1
CC	146	F58C	1	KISC	1	HEAT	14	CNMP	1	HECT	1	FHA	2
CH	3	FCH	1	LZ	2	HELIC	1	COLL	1	HOLI	1	FN3	4
CLH	7	FH	1	NES	3	HLH	3	CYCLIN	1	HR1	1	FU	3
CNMP	1	FHA	4	NLS	3	HOLI	4	DEXDc	9	I_Phosphatase	1	FYVE	1
CPDc	1	FN3	10	PAC	1	HOX	1	DSRM	1	IG	1	GoLoco	1
CYCLIN	1	FU	3	PB1	2	HRM	1	EGF	4	IPT	3	GuKinase	3
DEXDc	4	G_PATCH	1	PDZ	1	IG	4	EGFCA	2	KELCH	9	HELIC	2
DISINT	2	GTP_EFTU_D2	1	PH	9	IPT	3	EGFL	15	KISC	2	HLH	3
DSRM	2	GuKinase	3	PHD	1	KISC	4	F58C	1	KUNITZ	1	HOLI	4
EF	8	HAT	26	PISC	1	LA	1	FCH	1	LIM	20	IPT	5
ERM	2	HDc	1	PSI	1	LIM	20	FF	5	LZ	5	KISC	3
FCH	1	HEAT	1	PTBI	3	LZ	1	FH	2	NES	5	LA	1
FH	3	HECT	1	RA	1	MATH	1	FHA	4	NLS	8	LRR	1
FHA	3	HELIC	3	RAS	1	NES	5	FN3	6	NPXY	1	LZ	1
FN3	7	HLH	2	RBD	2	NLS	8	FU	1	PB1	1	NES	4
FU	4	IG	4	REC	4	PAC	1	FYVE	1	PBD	2	NLS	4
HEAT	19	IGC2	1	RGS	1	PAS	3	G_PATCH	1	PbH1	5	PAS	2
HELIC	4	IL6	1	RHOD	2	PB1	6	GEL	4	PDZ	34	PB1	2
HMG	1	IPT	3	RHOGAP	1	PBD	1	GTP_EFTU_D	1	PH	16	PBD	2

HR1	1	KISC	4	RHOGEF	6	PDZ	17	GuKinase	2	PHD	1	PDZ	11
HSF	1	LA	1	RING	2	PH	9	HEAT	1	PI3KA	1	PEST	1
IBN_NT	4	LIM	1	S_T_kinase	19	PHD	1	HECT	1	PI3KC	2	PH	10
IL6	1	LRR	7	S_T_Y_Kinase	2	PI3KA	1	HELIC	9	PI3KC2	1	PHD	1
IPT	4	LZ	6	SEMA	1	PI3KC	1	HLH	1	PRY	1	PI3KA	3
IQ	4	MA3	1	SH2	6	PP2A	1	I_Phosphatase	2	PSI	1	PI3KC	5
JAB	1	MIF4G	1	SH3	10	PSI	1	IBN_NT	1	PTB	1	PI3KC2	2
KH	4	MORN	8	SORB	1	PTB	10	IG	1	PTBI	2	PSI	2
KISC	3	NES	9	SP	12	PTBI	2	IGC2	1	PX	1	PTBI	1
Ku78	1	NLS	12	SYNUC	1	RA	5	IGFLMN	24	RA	1	RA	2
LA	1	NPXY	1	TGFB	1	RAS	1	IL6	1	RasGEF	1	RAS	1
LIM	2	PB1	4	TM	59	RBD	4	KISC	5	RBD	4	RasGEF	1
LISH	1	PBD	1	Tyr_Kinase	7	RHO	1	LA	1	REC	2	RasGEFN	1
LRR	19	PDZ	23	UBA	5	RHOD	2	LDLA	31	RHD	2	RBD	2
LZ	5	PEST	2	UCH	1	RHOGAP	2	LDLRB	34	RHO	1	REC	2
MA3	1	PH	13	VPS9	1	RHOGEF	4	LIM	16	RHOD	1	RGS	2
MATH	1	PHD	1	WD40	11	RING	2	LISH	1	RHOGAP	4	RHOD	1
MIF4G	1	PI3KC	2	WW	1	S_T_kinase	20	LRR	12	RHOGEF	5	RHOGAP	1
NES	5	PSI	3	ZnF_RBZ	2	S_T_Y_Kinase	1	LZ	7	RING	6	RHOGEF	3
NLS	26	PTBI	3	ZnFA20	7	SEMA	1	MCM	1	RRM	3	RING	1
PB1	5	RA	4			SH2	4	MIF4G	1	S_T_kinase	15	RRM	6
PBD	1	RAS	2			SH3	10	NES	9	S_T_Y_Kinase	2	S_T_kinase	13
PDZ	11	RASGAP	1			SM	1	NLS	14	SAM	12	S_T_Y_Kinase	1
PEST	2	RasGEF	1			SP	8	PB1	3	SAP	2	SAM	4
PGK	1	RBD	3			TBC	5	PBD	2	Sec7	1	SAP	1
PH	11	REC	4			TM	44	PbH1	5	SEMA	1	SEMA	1
PHD	1	RGS	2			TPR	28	PDZ	20	SH2	7	SET	1
PI3KA	1	RHD	2			Tyr_Kinase	5	PH	28	SH3	16	SH2	2
PI3KC	2	RHOD	3			Tyr_Phos	2	PHD	1	SORB	1	SH3	5
PI3KC2	1	RHOGAP	3			UBA	3	PI3KA	1	SP	12	SP	12
PINT	1	RHOGEF	5			UBQ	1	PI3KC	3	SPRY	2	STI1	3
PLEC	14	RING	3			UCH	2	PI3KC2	1	TM	29	TM	69
PP2A	2	RRM	5			VHP	5	PP2C	1	TPR	7	TPR	28
PROF	1	S_T_kinase	23			WD40	5	PRY	1	Tyr_Kinase	6	TUBULIN	2
PRY	2	S_T_Y_Kinase	6			ZnF_C3H1	2	PTB	11	Tyr_Phos	4	Tyr_Kinase	3
PSI	1	SAM	4			ZnF_C4	4	PTBI	3	UBA	3	UBA	4
PTB	4	SAP	2			ZnF_RBZ	1	PWI	1	UCH	2	UBQ	1
PTBI	3	Sec7	1			ZnF_ZZ	1	RA	5	VHP	5	UCH	1
PX	1	SEMA	1			ZnFA20	7	RASGAP	1	WD40	24	VWA	1

RA	2
RAS	2
RASGAP	1
RasGEF	2
RasGEFN	1
RBD	3
REC	4
REP	2
RHD	2
RHO	1
RHOD	2
RHOGAP	3
RHOGEF	5
RING	5
RRM	16
S_100	2
S_T_kinase	22
S_T_Y_Kinase	1
S1	1
SAM	4
SANT	2
SAP	1
SEMA	1
SH2	2
SH3	5
SIS	1
SP	25
SPECTRIN	25
SPRY	2
TBC	2
TM	74
TPR	7
TUBULIN	3
Tyr_Kinase	3
Tyr_Phos	1
UBA	5
UBQ	9
VPS9	1
VWA	1
WD40	29
WSXWS	2

SH2	8
SH3	11
SP	15
SYNUC	1
TM	87
TPR	21
TUBULIN	1
TUDOR	1
Tyr_Kinase	8
Tyr_Phos	4
UBA	4
UBQ	9
UCH	1
VPS9	4
VWA	2
WD40	26
ZnF_RBZ	1
ZnFA20	9
ZNFC2	2

RasGEF	2
RasGEFN	1
RBD	7
REC	2
RGS	1
RHD	2
RHOD	1
RHOGAP	4
RHOGEF	12
RING	5
RRM	35
S_T_kinase	27
S_T_Y_Kinase	4
SAM	11
SANT	2
SAP	1
Sec7	1
SET	1
SH2	5
SH3	17
SP	12
SPECTRIN	17
SPRY	1
TBC	4
TM	37
TPR	24
TUBULIN	4
TUDOR	1
Tyr_Kinase	4
Tyr_Phos	4
UBA	4
UBOX	1
UBQ	11
UCH	1
UIM	3
VHP	5
VPS9	1
WD40	27
WSXWS	1
WW	8
ZnF_C3H1	1

WW	7
WWE	2
ZnF_C4	1
ZnF_RBZ	1
ZNFC2	4

WD40	19
WW	4
ZnF_C4	4

WW	1				ZnF_RBZ	3		
ZnF_C3H1	1				ZnFA20	8		
ZnFA20	7				ZNFC2	23		
ZNFC2	3							
Total	746	642	313	488		1049	458	442
