# Supplementary material for: Human 14-3-3 Paralogs Differences Uncovered by Cross-Talk of Phosphorylation and Lysine Acetylation
Source: PLoS One. 2013 Feb 13;8(2):e55703. doi: 10.1371/journal.pone.0055703 (PMC3572099; doi:10.1371/journal.pone.0055703)
Supplement: Table S5 — Fisher exact test ( p -values) results of number of acetylated partners from each 14-3-3 paralog. (PDF) [file pone.0055703.s011.pdf]

Table 5. Fisher exact test (*p*-values) results of number of acetylated partners from each paralogs.

14-3-3 isoform compared to zeta	*p*-value (Fisher exact test)
beta	0.0002342
gamma	0.002426
sigma	$1.652e^{-10}$
eta	0.0001431
epsilon	$8.645e^{-05}$
theta	0.0004458
