# Supplementary material for: Human 14-3-3 Paralogs Differences Uncovered by Cross-Talk of Phosphorylation and Lysine Acetylation
Source: PLoS One. 2013 Feb 13;8(2):e55703. doi: 10.1371/journal.pone.0055703 (PMC3572099; doi:10.1371/journal.pone.0055703)
Supplement: Table S6 — Fisher exact test ( p -values) results of number of each modified amino acid from 14-3-3 paralogs. (PDF) [file pone.0055703.s012.pdf]

Table 6: Fisher exact test results for the modification of serine, threonine, tyrosine and lysine residues of each 14-3-3 isoform network

		pS	npS	totals	*p*-value	pT	npT	totals	*p*-value	pY	npY	totals	*p*-value
Beta	acK	49	3	52	$< 2.2e^{-16}$	46	6	52	$4.36e^{-15}$	36	16	52	0.003508
	nacK	92	6	98		73	25	98		42	56	98	
	totals	141	9	150		119	31	150		78	72	150	
Epsilon	acK	22	3	25	$1.511e^{-13}$	21	4	25	$7.09e^{-10}$	15	10	25	0.0997
	nacK	47	5	52		42	10	52		21	31	52	
	totals	69	8	77		63	14	77		36	41	77	
Eta	acK	35	6	41	$< 2.2e^{-16}$	30	11	41	$8.24e^{-10}$	13	28	41	0.5922
	nacK	75	5	80		59	21	80		33	47	80	
	totals	110	11	121		89	32	121		46	75	121	
Gamma	acK	106	6	112	$< 2.2e^{-16}$	97	15	112	$< 2.2e^{-16}$	68	44	112	0.01155
	nacK	163	11	174		130	44	174		75	99	174	
	totals	269	17	286		227	57	286		143	143	286	
Sigma	acK	22	3	25	$< 2.2e^{-16}$	19	6	25	$8.0e^{-13}$	17	8	25	$1.66e^{-05}$
	nacK	85	5	90		60	30	90		40	50	90	
	totals	107	8	115		79	36	115		57	58	115	
Theta	acK	32	2	34	$< 2.2e^{-16}$	26	8	34	$1.33e^{-08}$	16	18	34	0.4614
	nacK	58	6	64		48	16	64		24	40	64	
	totals	90	8	98		74	24	98		40	58	98	
Zeta	acK	146	21	167	$< 2.2e^{-16}$	128	39	167	$1.82e^{-09}$	93	74	167	0.4342
	nacK	148	12	160		114	46	160		63	97	160	
	totals	294	33	327		242	85	327		156	171	327	
